# Novel fluorescent probes for the fluoride anion based on hydroxy-substituted perylene tetra-(alkoxycarbonyl) derivatives†

**DOI:** 10.1039/c8ra00299a

**Published:** 2018-04-16

**Authors:** Fengxia Zhang, Yunlong Zhao, Yanhui Chi, Yongshan Ma, Tianyi Jiang, Xiaofeng Wei, Qian Zhao, Zhiqiang Shi, Jingmin Shi

**Affiliations:** College of Chemistry, Chemical Engineering and Materials Science, Collaborative Innovation Center of Functionalized Probes for Chemical Imaging in Universities of Shandong, Key Laboratory of Molecular and Nano Probes, Ministry of Education Shandong Provincial Key Laboratory of Clean Production of Fine Chemistry, Shandong Normal University Jinan 250014 P. R. China zshi@sdnu.edu.cn shijingmin1955@163.com; Shandong Provincial Key Laboratory of Metrology and Measurement, Shandong Institute of Metrology, Shandong Social Justice Institute of Metrology Jinan 250014 P. R. China; School of Municipal and Environmental Engineering, Shandong Jianzhu University Jinan 250101 P. R. China mlosh@sdjzu.edu.cn; Co-Innovation Center of Green Building Jinan 250101 P. R. China

## Abstract

The fluoride anion (F^−^) sensing abilities of 1-hydroxyl-3,4,9,10-tetra (*n*-butoxyloxycarbonyl) perylene (probe 1) and 1-hydroxyl-mono-five-membered *S*-heterocyclic annulated tetra (*n*-butoxyloxycarbonyl) perylene (probe 2) were studied through visual detection experiments, UV-Vis, fluorescence, and ^1^H NMR titrations. The probes were sensitive and selective for distinguishing F^−^ from other anions (Cl^−^, Br^−^, I^−^, SO_4_^−^, PF_6_^−^, H_2_PO_4_^−^, BF_4_^−^, ClO_4_^−^, OH^−^, CH_3_COO^−^, and HPO_4_^2−^) through a change of UV-Vis and fluorescence spectra. The absorption and fluorescence emission properties of the probes arise from the intermolecular proton transfer (IPT) process between a hydrogen atom on the phenolic O position of probe and the F^−^ anion. The sensing mechanism was supported by theoretical investigation. Moreover, probe-based test strips can conveniently detect F^−^ without any additional equipment, and they can be used as fluorescent probes for monitoring F^−^ in living cells.

## Abstract 2

The fluoride anion (F^−^) sensing abilities of two fluorescent probes based on hydroxy-substituted perylene tetra-(alkoxycarbonyl) derivatives were studied through visual detection experiment, UV-Vis, fluorescence, and ^1^H NMR titrations.

## Graphical abstract

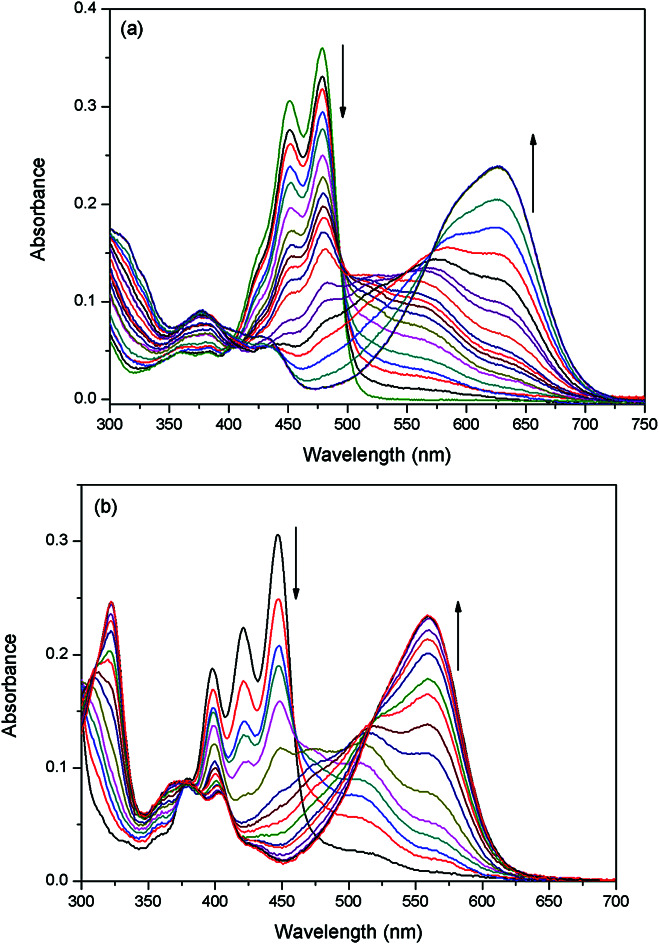

## Introduction

The fluoride anion (F^−^) has duplicitous effects in biological systems and the environment.^1–3^ While it exhibits a beneficial effect in preventing dental caries and has been widely adopted in the treatment of osteoporosis, excessive intake of it by the human body may cause fluorosis, urolithiasis, and even cancer.^4–8^ In this context, chemosensors that are capable of detecting F^−^ efficiently and selectively have aroused growing interest in the past decades.^9–11^ A large number of fluorescent probes for F^−^ with various chemical structures have been developed.^12–14^ Also, different working models that based on the changes of color, electrochemical property, UV-Vis absorption, or fluorescence emission have been established.^15–17^

Perylene diimides (PDIs) have been widely used as industrial pigments.^18,19^ Their applications as materials for fluorescent probe have drawn considerable attention in recent years.^20,21^ These probe usually have good photostability, high thermal stability, high fluorescence quantum yield, and excellent chemical inertness.^22,23^ However, PDIs display poor solubility and very weak fluorescence owing to the aggregation of perylene chromophores, which limits their applications.^24,25^ Perylene tetra-(alkoxycarbonyl) (PTAC) is structurally related to PDIs but has four electron deficient carboxylic ester chains connected to the perylene core. It shows excellent fluorescence and good solubility in organic solvents.^26,27^ Developing core-extended PDIs is a good approach to improve the electronic and optical properties of the PDIs.^28^ Incorporating heteroatom into the perylene skeleton has been extensively explored, and a large number of PDI derivatives decorated with diverse hetero cycles such as S^−^, O^−^, and N^−^ hetero cycles in the bay regions have been demonstrated.^29,30^

**Scheme 1:**
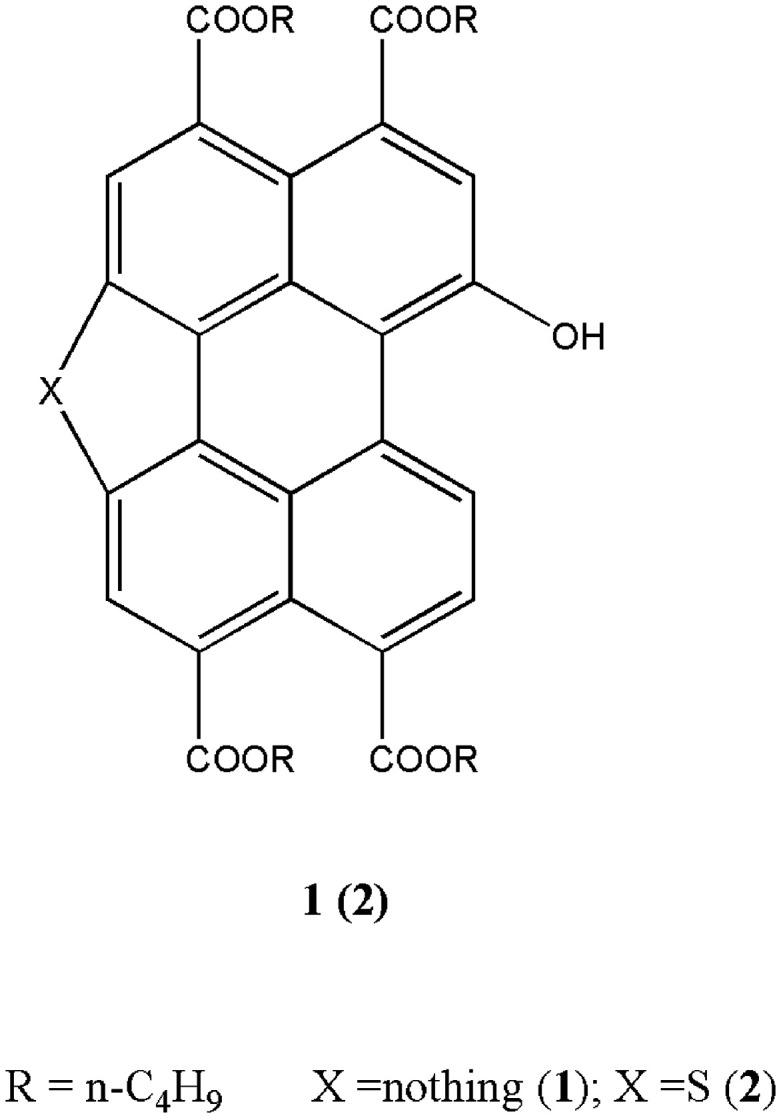
Structure of 1-hydroxyl-3,4,9,10-tetra (*n*-butoxyloxycarbonyl) perylene (1) and 1-hydroxyl-mono-five-membered *S*-heterocyclic annulated tetra (*n*-butoxyloxycarbonyl) perylene (2).

Among various F^−^ probe developed in the past decades, the use of NH unit, including urea/thiourea, imidazole, indole, pyrrole, amine, and amide, to bind the F^−^*via* H-bond interaction, have been thoroughly investigated.^31,32^ Recently, we have reported PTAC base probes for selectively sensing of F^−^ in which the H-bond donor chloroacetamide fragment is directly attached to electron deficient perylene moiety (which also serves as the optical signaling unit).^33^ An intermolecular proton transfer process between a hydrogen atom on the amide N position of probe and the F^−^ plays a key role in their sensing properties. In contrast, although a hydroxyl group (OH) can also be an admirable H-bonding donor, and its proton even shows more acidity compared with the proton of NH unit, few of attention has been paid to OH based system.^34,35^ It can be ascribed to the low recognition selectivity of OH when it binds to F^−^ through the formation of H-bond.^36^ In continuation of these studies, we report two novel OHs modified PTACs which display higher degree of F^−^ discrimination by direct deprotonation of OH unit (Scheme 1). The visual detection observation indicated higher sensitivity of the new probe with OH unit (6 equiv. of F^−^ for probe 1 and 5 equiv. of F^−^ for probe 2, respectively) towards F^−^ ion than the reported probes with NH unit (8 equiv. of F^−^ for probe 1 and 10 equiv. of F^−^ for probe 2, respectively). The absorption and fluorescence emission properties of the probes arise from the intermolecular proton transfer (IPT) process between a hydrogen atom on the phenolic O position of probe and the F^−^ anion. Test strips were developed based on probes 1 and 2 for instant sensing of F^−^, and the probe were further applied for the detection of F^−^ in living cells.

## Results and discussion

### Absorption spectral response toward F^−^

**Fig. 1:**
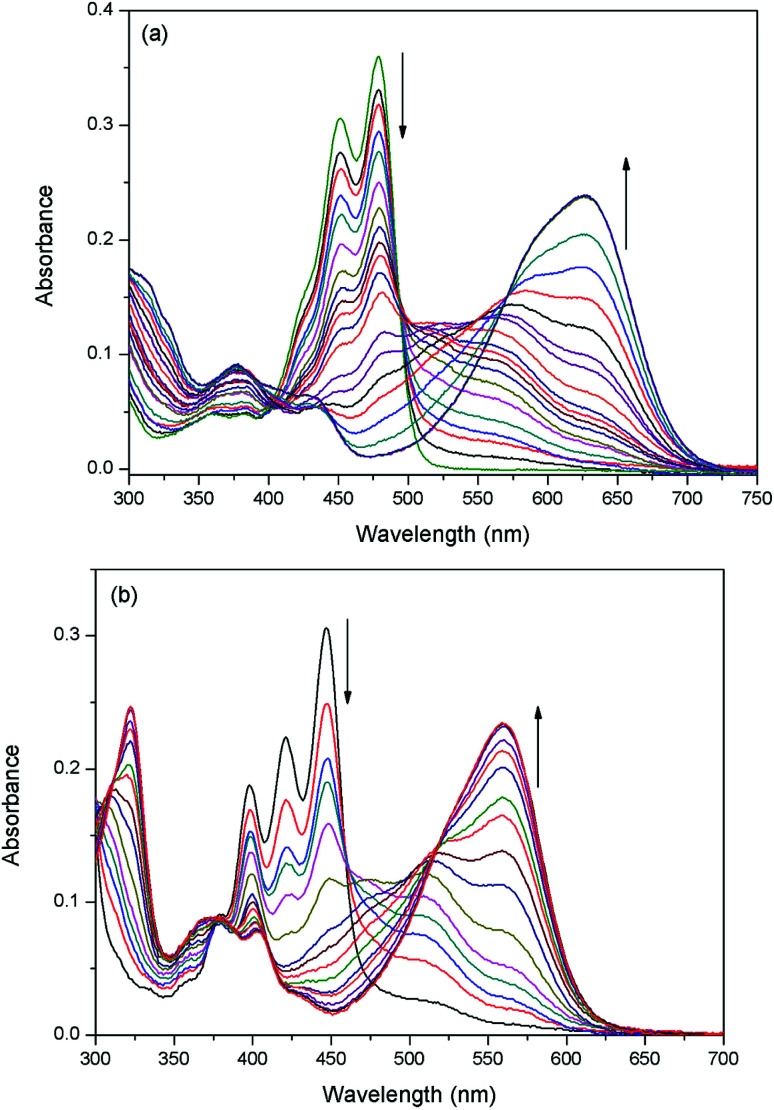
(a) UV-Vis absorption spectra of compound 1 (10 μM) in CH_2_Cl_2_ upon addition of 0–6 equivalent of F^−^ anions. (b) UV-Vis absorption spectra of compound 2 (10 μM) in dichloromethane upon addition of 0–5 equivalent of F^−^ anions.

The sensing properties of compounds 1 and 2 to F^−^ were examined in dichloromethane (CH_2_Cl_2_) solution by adding tetrabutylammonium salt of F^−^ (TBAF). A gradual spectral change is shown in Fig. 1. Probe 1 exhibited an absorption maximum centered at 485 nm and a small shoulder at 459 nm. After the addition of F^−^ (from 0 to 1.0 equiv.) to solution 1, the intensity bands at 459 nm and 485 nm (*ε* = 36 122 L mol^−1^ cm^−1^) gradually decreased with the appearance of new bands at 564 nm and 627 nm. In the presence of 6 equiv. of F^−^, the bands at 564 nm and 627 nm predominated with only one clear isosbestic point.

The absorption peaks of probe 2 appeared at 447 nm (*ε* = 30 622 L mol^−1^ cm^−1^) and 421 nm. Compared to probe 1, they were blue-shifted (∼38 nm) because of the extended aromatic core along the short molecular axis.^37^ Probe 2 also revealed the above mentioned phenomena of probe 1 when F^−^ was added. Adding F^−^ (from 0 to 1.0 equiv.) to solution 2 leaded to the appearance of absorption bands at 510 nm and 560 nm at the expense of absorption quenching at 447 nm and 421 nm.

UV-Vis titrations of 1 (2) have demonstrated three clear isosbestic points at 403 nm, 493 nm and 571 nm (379 nm, 459 nm and 517 nm for 2), which indicated that they should be transformed to two new species with the addition of F^−^. The observed new bands at long wave suggest that a deprotonation process could be involved. According to Job's plot (Fig. S-1†), probe and F^−^ formed 1 : 1 stoichiometry complexes. This is because the naphthol group contains one relatively acidic OH group as a rigid binding site with F^−^. In most cases, the relatively strong base F^−^ can deprotonate the acidic phenolic OH group to form weak acid HF (p*K*_a_ = 1.5). In particular, the responses of two probes to F^−^ were both very fast, normally within 1 s, revealing the probes were real-time.

### Fluorescence response toward F^−^

**Fig. 2:**
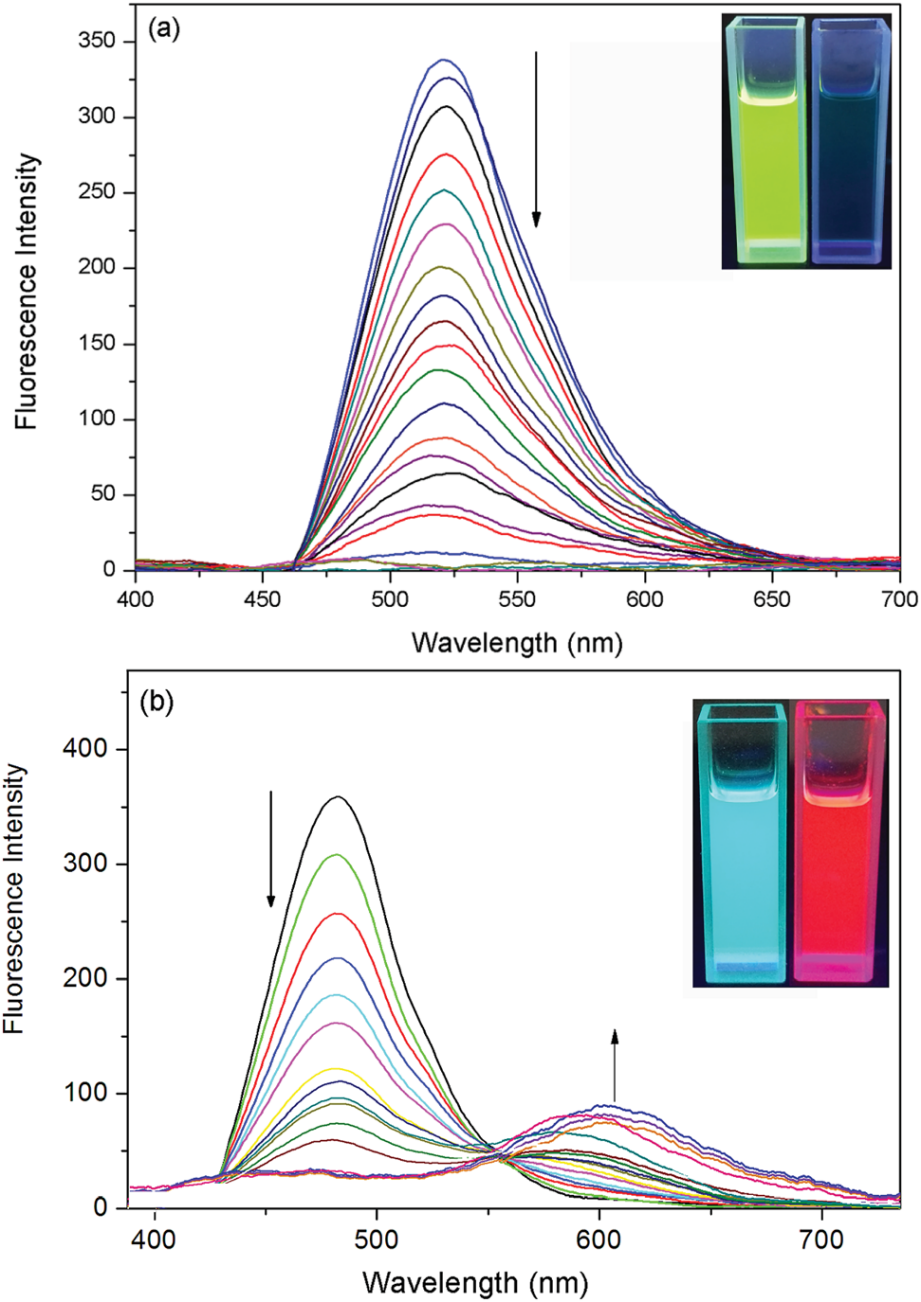
(a) Fluorescence titration spectra (*λ*_ex_ = 571 nm) of probe 1 (10 μM) in CH_2_Cl_2_ upon addition of 0–6 equivalent of F^−^ anions. (b) Fluorescence titration spectra (*λ*_ex_ = 517 nm) of probe 2 (10 μM) in CH_2_Cl_2_ upon addition of 0–5 equivalent of F^−^ anions. The inset shows F^−^ dependent photographs under UV light (*λ*_ex_ = 365 nm).

The fluorescence titration of the probes with F^−^ were carried out. As shown in Fig. 2a, the addition of incremental concentration (0–6 × 10^−5^ M) of F^−^ to the strong fluorescent DCM solution of the probe 1 caused a gradual fluorescence decrease at 517 nm. As a result, an obvious color change from bright green to faint blue under the irradiation of a UV-lamp was clearly observed (Fig. 2a inset). Furthermore, a corresponding correlation between emission response of probe 1 at 517 nm and F^−^ concentration (0–2 × 10^−5^ M) in DCM was obtained in Fig. S-2a.† The linear equation was *y* = −155.65*x*+313.71, in which *x* was the concentration of F^−^ (<2 × 10^−5^ M) and *y* was the fluorescence intensity of 489 nm. The coefficient of determination *R* was 0.973. The detection limit was evaluated to be 0.48 μM (as three times the standard deviation of the blank signal).^38^

The probe 2 showed a maximum fluorescence emission peak at 489 nm without F^−^. However, with the addition of incremental concentration (1 × 10^−5^ to 5 × 10^−5^ M) of F^−^, a new band at 597 nm appeared (Fig. 2b). It was because the strong molecular planarity conformation provided efficient π-conjugation and therefore favored the efficient ICT from the electron donating (O^−^) moiety to the electron accepting groups. Additionally, the emission intensity (*I*_489_) decreased linearly along with the F^−^ concentration from 0 to 2 × 10^−5^ M (Fig. S-2b†). The linear equation was *y* = −101.68*x* + 207.16 and the coefficient of determination *R* were 0.981. The estimated detection limit using the fluorescence titration data was evaluated to be 0.55 μM, which was higher than that of probe 1. With respect to the spectra changes, an obvious fluorescence color change from sky-blue to red was observed.

### Sensing mechanism of probe toward F^−^

OH group demonstrated highly acidic hydrogen-bond donor. Due to the highly electron-deficient arene characterization of PDIs, its acidity will be strengthened if directly attached to PDIs.^39^ Another, the high basicity of F^−^ in organic solvents can lead to deprotonation of OH group which often results in dramatic absorption and fluorescence change.^40^

**Fig. 3:**
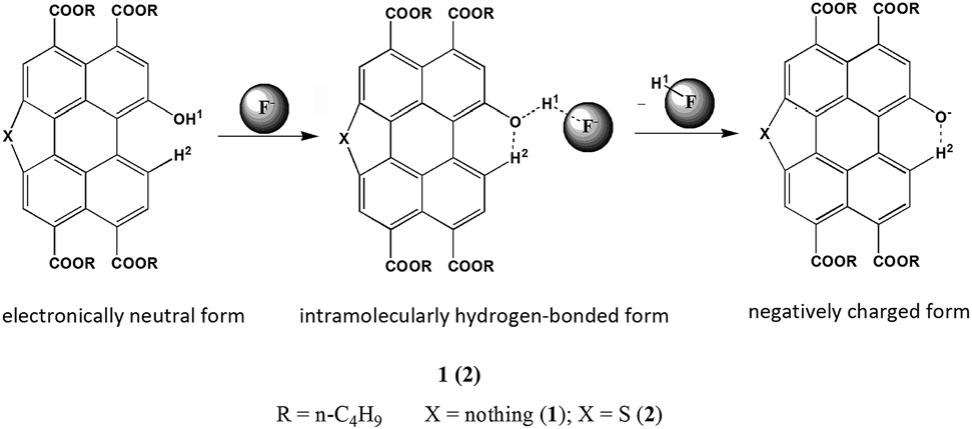
Three forms of the probes.

We designed the probes that could bind H^+^ of OH unit in the perylene skeleton with F^−^ to determine the level of F^−^. The F^−^ sensitivity is introduced *via* the OH unit upon either protonation or deprotonation. The probes existed in either electronically neutral form (1 or 2: PTACs-OH) without a F^−^ or negatively charged form (1^−^ or 2^−^: PTACs-O^−^) with a F^−^. The perylene skeleton had nearly planar conformation, and the phenolic O^−^ group could be easily intramolecularly hydrogen-bonded with the adjacent H atoms of perylene core. Between neutral and negatively charged, there is another form: the hydrogen-bonding complex form (Fig. 3). This planar conformation provided efficient π-conjugation and four *n*-butoxyloxycarbonyl groups were also attached to act as electron-accepting groups. These facilitated the efficient intramolecular charge transfer transition (ICT) from the electron donating (O^−^) moiety to the electron accepting groups. The presence of two isosbestic points (496 nm and 571 nm for probe 1, 461 nm and 517 nm for probe 2) in the absorption spectra is due to the formation of two forms (the negatively charged form and the intramolecularly hydrogen-bonded form). In the negatively charged form and the intramolecularly hydrogen-bonded form, probes exhibit weak emission due to strong electron-donating groups of O^−^.

**Fig. 4:**
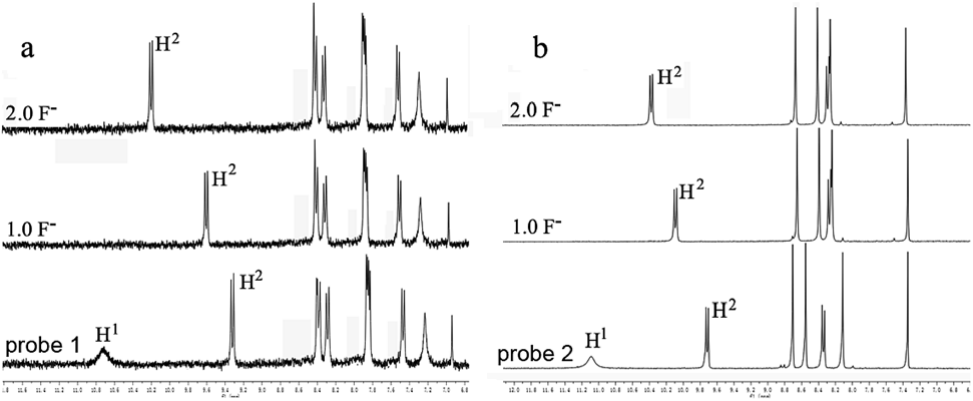
NMR titration of (a) probe 1 and (b) probe 2 with TBAF in CDCl_3_ at 298 K. Conditions: [probes] = 1.0 × 10^−2^ M, [TBAF] = 0–2.0 × 10^−2^ M.

To further understand the interaction between TBAF and receptor, ^1^H NMR titration experiments were carried out in chloroform (CDCl_3_). Fig. 4a and b shows the partial ^1^H NMR spectra of probe 1 (2) upon addition of F^−^. As can be seen, in the electronically neutral form, probe 1 (2) showed resonance for the OH proton at 10.68 ppm (11.09 ppm). Other aromatic protons of probes appeared at their usual positions in the range from 6.9 ppm to 10.3 ppm. With the addition of 1.0 equiv. of F^−^ to probe 1 (2), the OH signal disappeared and the H^2^ proton signal change from 9.33 ppm to 9.62 ppm (9.72 ppm to 10.12 ppm) due to the formation of intramolecular hydrogen bonds between the phenolic O^−^ and adjacent H atoms of perylene core. The probes existed in the hydrogen-bonding complex form. With the addition of F^−^ beyond 2 equiv., probes were gradually changed to negatively charged form. This suggests that F^−^ selective optical probes relies on deprotonation mechanism that occurrs between F^−^ and hydroxyl group.

### Selectivity investigation

**Fig. 5:**
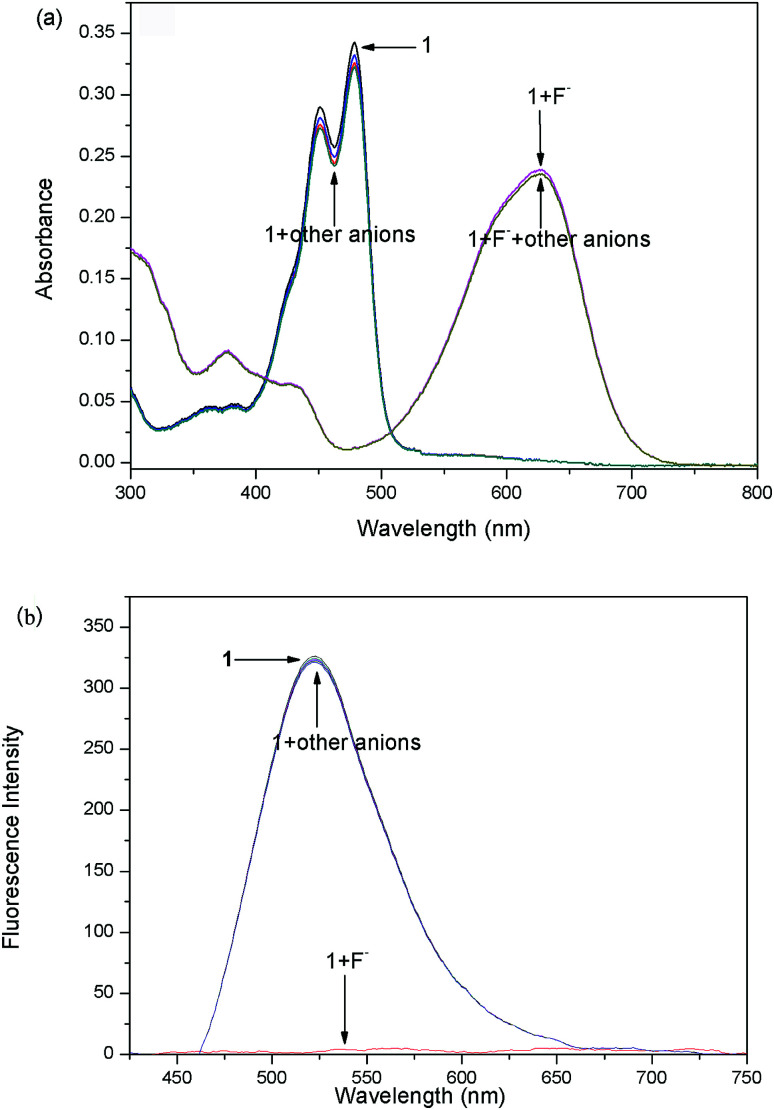
Absorption (a) and fluorescence (b) spectra of probe 1 (10 μM) in CDCl_3_ solution upon the addition of 6 equiv. of various anions (tetrabutylammonium salts) and ammonium hydroxide.

**Fig. 6:**
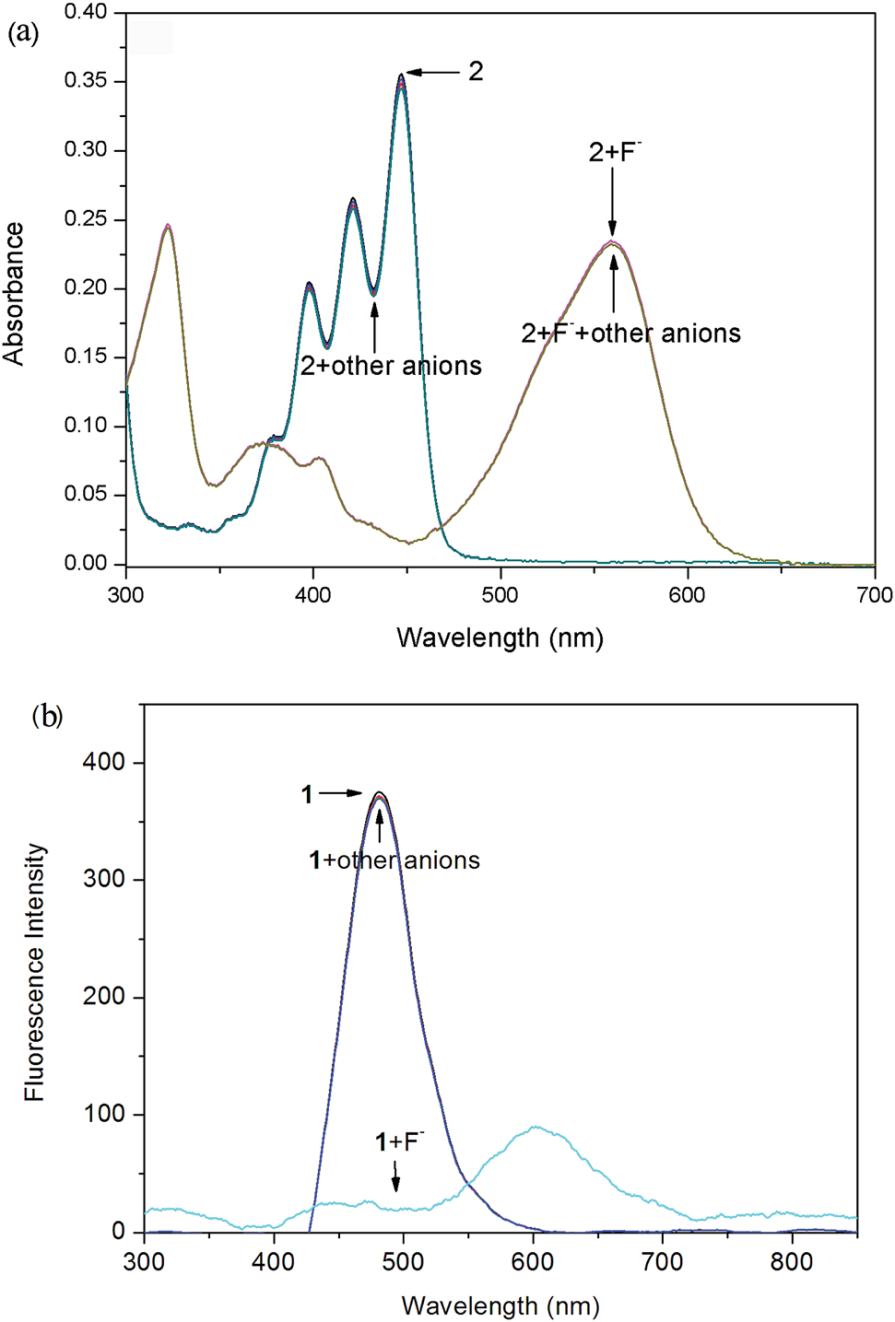
Absorption (a) and fluorescence (b) spectra of probe 2 (10 μM) in CDCl_3_ solution upon the addition of 5 equiv. of various anions (tetrabutylammonium salts) and ammonium hydroxide.

In order to check the interference of other anions to the detection of F^−^, the absorption and fluorescence spectra of probes 1 (Fig. 5) and 2 (Fig. 6) were recorded in the presence of other competitive anions. The results indicated that the absorbance and fluorescence spectra of both 1 and 2 did not show significant change in the presence of Cl^−^, Br^−^, I^−^, SO_4_^−^, PF_6_^−^, H_2_PO_4_^−^, BF_4_^−^, ClO_4_^−^, CH_3_COO^−^, HPO_4_^2−^ (as salt of tetra-*n*-butylammonium) and OH^−^ (ammonium hydroxide). Also, the absorbance and fluorescence intensity of the probe–F^−^ complex showed no obvious change in the presence of other anions. The high basicity and electronegativity of F^−^ anion may be accounted for its selectivity to various anions. This suggested that the probes can recognize F^−^ through absorption, and fluorescence spectral methods.

### Density functional theory (DFT) simulations

**Fig. 7:**
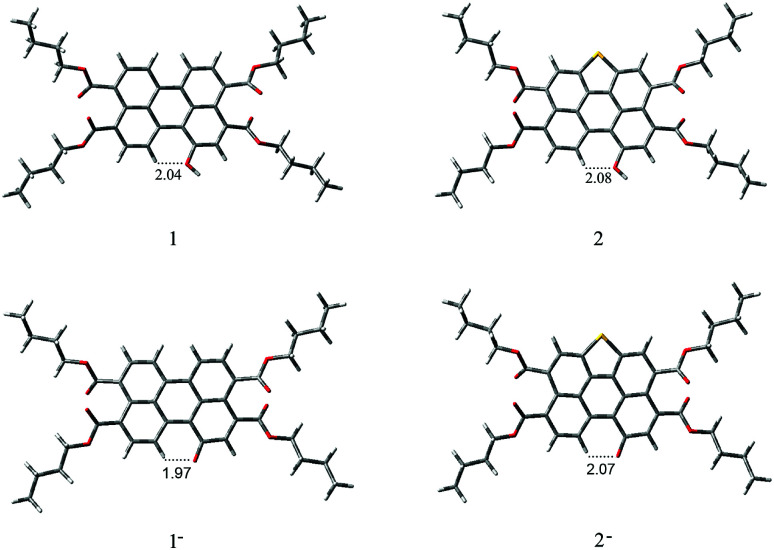
Geometry-optimized structures of 1, 1^−^, 2 and 2^−^.

**Fig. 8:**
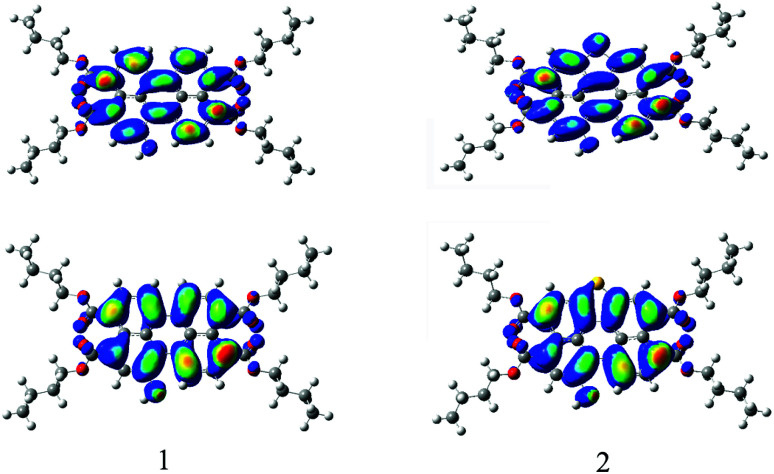
Computed Frontier orbitals of 1 and 2. The upper graphs are the LUMOs and the lower ones are the HOMOs.

To gain insight into the electronic structure properties of probes 1 and 2, energy-optimized structures of probes (1, 2) and their corresponding negatively charged forms (1^−^, 2^−^) were obtained on DFT calculations (Fig. 7).^41,42^ The spatial distributions and orbital energies of HOMOs and LUMOs were also generated based on these calculations (Fig. 8).

According to the calculations, the phenolic O^−^ groups are intramolecularly hydrogen-bonded with the adjacent H atoms of perylene core. The distances between the O atom and H atom of 1, 1^−^, 2, and 2^−^ are 2.04 Å, 1.97 Å, 2.08 Å, and 2.07 Å, respectively. Such short bond lengths suggest that these intramolecular hydrogen bonds are quite strong and thus might promote the OH units from forming intermolecular hydrogen bond with F^−^. Furthermore, both of the ground-state geometries of 1, 1^−^, 2, and 2^−^ had small core twist angles because of the introduction of O–H moiety at the bay position. The approximately dihedral angles between the two naphthalene subunits attached to the central benzene ring of 1, 1^−^, 2 and 2^−^ are 1.02°, 1.69°, 0.61°, and 0.45°, respectively. All molecules of the perylene skeleton and the phenolic O^−^ groups of 1^−^ and 2^−^ had nearly planar conformation, which provided effective π-conjugation in the fused system and therefore facilitated the efficient ICT transition from the electron-donating (O^−^) moiety to the electron-accepting groups, and resulted in a substantial red shift in the absorption/emission spectrum.

As the results shown, both HOMO and LUMO of 1 delocalized over whole perylene molecule and esters systems evidently including hydroxyl group. By comparison, the HOMO orbital of probe 2 was centered on the perylene ring and esters systems, and the LUMO orbital was extended from the central perylene core to the S atom site. The HOMO/LUMO energy of 1 and 2 were −5.22/−2.42 eV and −5.33/−2.34 eV, respectively. The calculated HOMO–LUMO gap of 2 (2.99 eV) was relatively higher than that of 1 (2.80 eV). This was consistent with the blue-shift of the electronic spectra of probe 2 in comparison with that of probe 1. From these results, we could conclude that the energy level of molecular orbitals as well as the HOMO–LUMO gap can be tuned by the five membered *S*-heterocyclic annulu.

### Cell cytotoxicity assays

The cytotoxicity of probes 1 and 2 on Human Lung Cancer A549 Cells were evaluated using MTT assay with the probes concentration from 0.01 μM to 50 μM^43^ (Fig. S-3†). The results demonstrate that both probes 1 and 2 have little toxicity and good biocompatibility to cultured cells. The low cytotoxicity could be ascribed to the four carboxylic ester chains which protect the dyes from interacting nonspecifically with the extracellular proteins and triggering antigenicity and immunogenicity inside the cells.^44^ This is an advantage for the utility of probes 1 and 2 in live cell imaging.

### Imaging of living cells

**Fig. 9:**
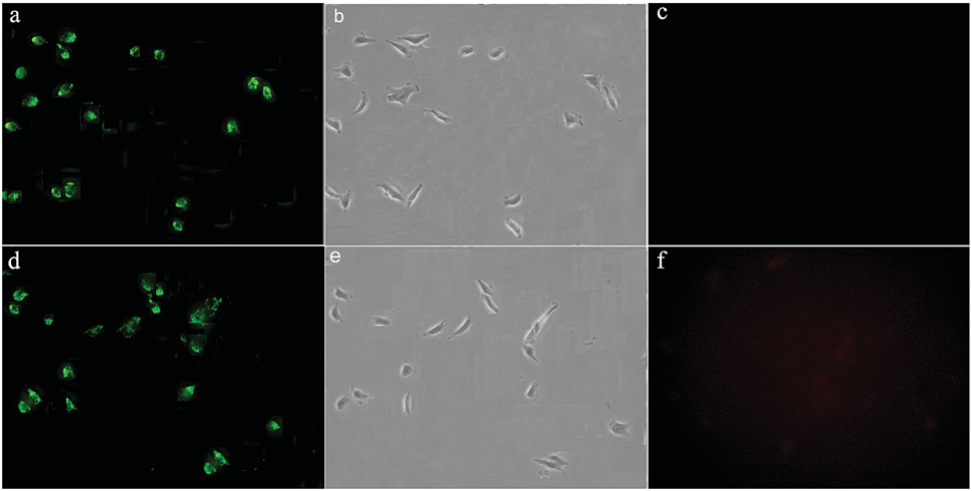
Live-cell imaging of F^−^ in A549 cells: (a) fluorescence image of cells incubation with probe 1 (10 μM) for 30 min at 37 °C (b) bright-field image of A549 cells (c) fluorescence image of A549 cells incubated with probe 1 (10 μM) and subsequently treated with NaF (60 μM) for 15 min (d) fluorescence image of cells incubation with probe 2 (10 μM) for 30 min at 37 °C (e) bright-field image of A549 cells (f) fluorescence image of A549 cells incubated with probe 2 (10 μM) and subsequently treated with NaF (60 μM) for 15 min.

The ability of the probes 1 and 2 for the detection of F^−^ in living cells was investigated using confocal fluorescence microscopic imaging. The A549 cells were incubated with probes 1 and 2 dissolved in DMSO (50 μM) at 37 °C for 30 min and then were imaged through confocal fluorescence microscopy with a blue-filter source of 460–490 nm. It reveals that the cells incubated with probes 1 and 2 displayed strong intracellular fluorescence (Fig. 9a and d). As judged from their morphology, the A549 cells appeared healthy and were viable after treated with the probe (Fig. 9b and e). The probe treated cells supplemented with 60 μM NaF in the medium with PBS buffer for 10 min at 37 °C. The cells incubated with probes 1 and 2 showed quenched fluorescence (Fig. 9c and f). These results indicate that probes 1 and 2 were good candidates for monitoring F^−^ changes in living cells.

### Test strips measurement

**Fig. 10:**
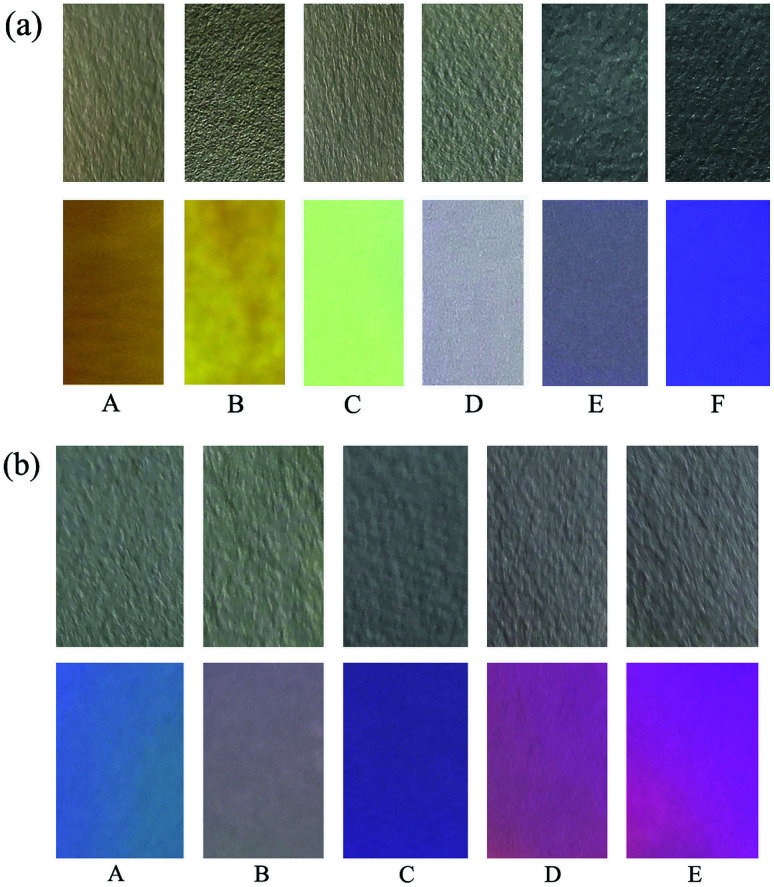
Photographs of test strips of 1 (a) and 2 (b) at various equiv. of F^−^ under ambient (top) and UV light (bottom): (A) 0; (B) 1.0; (C) 2.0; (D) 4.0; (E) 6.0 and (F) 8.0.

To investigate the practical application of the probes, test strips were developed for instant sensing of F^−^. As a representative case, a paste of probes 1 and 2 in DCM was coated on a Whatman filter paper strip and dried in air. As shown in Fig. 10, when the probe-based test strips were dipped in the F^−^ solutions with different concentrations, an instantaneous color change was observed under ambient and UV light. Consequently, probes 1 and 2 had excellent fluorescence sensing performance in the solid state, and the probe-based test strips can conveniently detect F^−^ without requiring any additional equipment.

## Conclusions

In summary, we have developed two novel probes based on PTAC for selective recognition of the F^−^. An IPT mechanism plays a key role in the sensing properties, and the mechanism is supported by ^1^HNMR. Additionally, the optical probes can be used for the ultrasensitive determination of F^−^ in live cells and the probe-based test strips can be easily fabricated and be used in efficient F^−^ test kits. While most F^−^ probe have mainly been constructed by choosing NH unit as the hydrogen-bonding donor to bond F^−^, this work suggests that the use of OH could also be a promising way to design probe for F^−^ detection. One advantage of using phenolic OH is that the deprotonation of OH unit usually results in multiplex responses of the probe due to the generation of the phenolic anion. Taking this advantage, more phenolic OH^−^ based F^−^ probe are expected in the near future.

## Experimental

### Materials and instrumental methods

All chemicals and solvents were of reagent grade and purchased from commercial source, directly used without further purification. ^1^H NMR and ^13^C NMR spectra were taken on a Bruker Advance 300 spectrometer in CDCl_3_ at room temperature. Mass spectra were recorded on a Bruker Maxis UHR-TOF MS spectrometer. FT-IR spectra were measured using a Bruker Tensor-27 spectrophotometer. UV-Vis absorption spectra were measured with a Varian CARY-50 spectrophotometer. Fluorescence spectra measurements were performed using Hitachi FL-4500 spectrofluorometer. Solutions of probes were typically 1 × 10^−5^ M for UV-Vis and emission studies. Tetrabutylammonium salts were used for anion studies. Fluorescent images were taken on Olympus BH2 fluorescence microscope.

### Anion sensor study

Stock solutions (1.0 × 10^−5^ M) of probes 1 and 2 were prepared in CH_2_Cl_2_, and a stock solution of the guest (1.0 × 10^−3^ M) was prepared by dissolving F^−^, Cl^−^, Br^−^, I^−^, SO_4_^−^, PF_6_^−^, H_2_PO_4_^−^, BF_4_^−^, ClO_4_^−^, CH_3_COO^−^, and HPO_4_^2−^ (as salt of tetra-*n*-butylammonium) in CH_2_Cl_2_. Another stock solution of OH^−^ (1.0 × 10^−3^ M) was prepared by dissolving tetrahydrofuran solution of ammonium hydroxide into CH_2_Cl_2_. The procedure for the UV-Vis and fluorescence studies involved making sequential additions of anionic guest using pipettes to 3 mL of the host stock solutions in the spectrometric cell.

### Computational details

Both structure optimization and the property calculations were performed by the Becke's three parameter gradient-corrected hybrid density function B3LYP method and the standard 6-31G* basis set with the Gaussian 03 program installed on a Windows PC.

### Test strips measurement

The test strips were prepared by immersing filter papers (2 × 1 cm^2^) in the DCM solution of probe (10 M) and subsequently drying them in air. The tetrabutylammonium salts of F^−^ stock solution was diluted to different concentrations with DCM, and then test strips coated with probe were immersed in solutions of F^−^ with different concentrations for colorimetric and fluorimetric response studies.

### Cell culture and fluorescence imaging

Human lung cancer A549 cells, obtained from the Chinese Type Culture Collection (Shanghai Institute of Cell Biology, Chinese Academy of Science, China), were grown in Dulbecco's modified Eagle's medium (DMEM) containing streptomycin/penicillin (100 U mL^−1^) and 10% fetal bovine serum (FBS) in a 5% CO_2_ atmosphere at 37 °C in a humidified incubator. When the cells had reached confluence, they were detached in trypsin solution, rinsed in DMEM, centrifuged at 1500 rpm for 3 min, and then resuspended and subcultured according to standard protocols. The culture medium was removed, and then the A549 cells are incubated with probes 1 and 2 (10 μM in DMSO buffered with PBS, pH = 7.54) in the culture medium for 30 min at 37 °C. After washing with PBS three times to remove excess probe, the cells are further incubated with F^−^ (60 mM in H_2_O) for 10 min at 37 °C and imaged with Olympus BH2 confocal fluorescence microscope at an excitation using a blue-filter source of 460–490 nm.

### Synthesis and characterization

The synthesis of probes 1 and 2 is shown in Scheme S-1.† 1-Hydroxyl-3,4,9,10-tetra (*n*-butoxyloxycarbonyl) perylene (probe 1) was synthesized according to the previous publication.^44^ Then compound 1 was dissolved in CH_2_Cl_2_. Fuming nitric acid was then added dropwise into the solution and the reaction mixture was kept stirring for 1 h at room temperature. Then a mixture of the crude product and sulfur powder was dissolved in anhydrous *N*-methyl pyrrolidine. The resulting solution was heated to 110 °C with vigorous stirring for 5 h, then cooled and poured into 2 M HCl. The precipitate was collected by vacuum filtration, washed with water and dried under vacuum condition. The residue was purified by gel column chromatography with dichloromethane/ethyl acetate (20/1) as eluent to afford target product 2 in 82% yields (see ESI† for details). Characterization data: ^1^H-NMR (CDCl_3_, 300 MHz, ppm): *δ* = 11.09 (s, 1H), 9.73 (d, *J* = 9.0 Hz, 1H), 8.71 (s, 1H), 8.55 (s, 1H), 8.33 (s, 1H), 8.11 (d, 1H), 4.34–4.43 (m, 8H), 1.74 (m, 8H), 1.48 (m, 8H), 0.88–0.99 (m, 12H). ^13^CNMR (75 MHz, CDCl_3_, ppm): *δ* = 169.59, 169.20, 168.81, 153.19, 129.87, 129.08, 128.16, 127.77, 126.94, 126.77, 125.43, 124.15, 123.01, 122.27, 119.10, 116.71, 65.71, 65.61, 65.42, 30.81, 29.67, 19.34, 13.84. FT-IR (KBr, cm^−1^): *ν* = 3365 (s, O–H stretching), 3189 (s, aliphatic C–H), 2922 (*vs.*, aliphatic C–H), 2854 (*vs.*, aliphatic C–H), 1651 (s, C=O), 1462 (s, aromatic C=C), 1410 (s, C–O), 1067, 873, 732, 661, 586, 528, 486, 437. HRMS: C_40_H_42_SO_9_ (M^+^ – H), calcd, 697.2352, found 697.2469.

## Conflicts of interest

The authors declare no competing financial interest.

## Supplementary Material

RA-008-C8RA00299A-s001

## References

[cit1] Baruah U., Gogoi N., Majumdar G., Chowdhury D. (2015). Carbohydr. Polym..

[cit2] Mahapatra A. K., Maji R., Maiti K., Adhikari S. S., Mukhopadhyay C. D., Mandal D. (2014). Analyst.

[cit3] Zhao Y., Li Y., Qin Z., Jiang R., Liu H., Li Y. (2012). Dalton Trans..

[cit4] Kaur P., Kaur S., Singh K. (2011). Talanta.

[cit5] Zhang G., Wang L., Cai X., Zhang L., Yu J., Wang A. (2013). Dyes Pigm..

[cit6] Li J., Chen H., Lin H., Lin H. (2009). J. Photochem. Photobiol., B.

[cit7] Satheshkumar A., El-Mossalamy E. H., Manivannan R., Parthiban C., Al-Harbi L. M., Kosa S., Elango K. P. (2014). Spectrochim. Acta, Part A.

[cit8] Yang X., Zheng L., Xie L., Liu Z., Li Y., Ning R., Zhang G., Gong X., Gao B., Liu C., Cui Y., Sun G., Zhang G. (2015). Sens. Actuators, B.

[cit9] Geddes C. D. (2001). Meas. Sci. Technol..

[cit10] Sokkalingam P., Lee C. H. (2011). J. Org. Chem..

[cit11] Formica M., Fusi V., Macedi E., Paoli P., Piersanti G., Rossi P., Zappia G., Orlando P. (2008). New J. Chem..

[cit12] Amatori S., Ambrosi G., Borgogelli E., Fanelli M., Formica M., Fusi V., Giorgi L., Macedi E., Micheloni M., Paoli P., Rossi P., Tassoni A. (2014). Inorg. Chem..

[cit13] Roy A., Datar A., Kand D., Saha T., Talukdar P. (2014). Org. Biomol. Chem..

[cit14] Cametti M., Rissanen K. (2009). Chem. Commun..

[cit15] Ambrosi G., Formica M., Fusi V., Giorgi L., Macedi E., Micheloni M., Paoli P., Pontellini R., Rossi P. (2011). Chem.–Eur. J..

[cit16] Ambrosi G., Formica M., Fusi V., Giorgi L., Guerri A., Micheloni M., Paoli P., Pontellini R., Rossi P. (2007). Chem.–Eur. J..

[cit17] Khanmohammadi H., Rezaeian K. (2014). RSC Adv..

[cit18] Aigner D., Freunberger S. A., Wilkening M. (2014). et al.. Anal. Chem..

[cit19] Aigner D., Dmitriev R. I., Borisov S. M., Papkovsky D. B., Klimant I. (2014). J. Mater. Chem. B.

[cit20] Zhang W., Gan S. Y., Li F. H., Han D. X., Zhang Q. X., Niu L. (2015). RSC Adv..

[cit21] Chen M., Ding Y., Gao Y., Zhu X., Wang P., Shi Z., Liu Q. (2017). RSC Adv..

[cit22] Hariharan P. S., Pitchaimani J., Madhu V., Anthony S. P. (2017). Opt. Mater..

[cit23] Li G., Zhao Y., Li J., Cao J., Zhu J., Sun X., Zhang Q. (2015). J. Org. Chem..

[cit24] Lakshmi P., Ojha D. (2014). J. Mol. Liq..

[cit25] Kim J., Choi J., Namgoong J., Kim S., Sakong C., Yuk S. (2015). et al.. J. Inclusion Phenom. Macrocyclic Chem..

[cit26] Zhan C., Jiang Y. Y., Yang M. Y., Lu L. H., Xiao S. Q. (2014). Chin. Chem. Lett..

[cit27] Gupta R. K., Pradhan B., Pathak S. K., Gupta M., Pal S. K., Sudhakar A. A. (2015). Langmuir.

[cit28] Zhao G. J., Han K. L. (2009). J. Phys. Chem. A.

[cit29] Li Y. J., Liu T. F., Liu H. B., Tian M. Z., Li Y. L. (2014). Acc. Chem. Res..

[cit30] Yu Y. W., Li Y. J., Qin Z. H., Jiang R. S., Liu H. B., Li Y. L. (2013). J. Colloid Interface Sci..

[cit31] Wang Y. F., Zhang L., Zhang G. J., Wu Y., Wu S. Y., Yu J. J., Wang L. M. (2014). Tetrahedron Lett..

[cit32] Dibakar K. M., Subhasish R., Ayan D., Arindam B. (2013). Chem. Phys. Lett..

[cit33] Ma Y. S., Zhao Y. L., Zhang F. X., Jiang T. Y., Wei X. F., Shen H., Wang R., Shi Z. Q. (2017). Sens. Actuators, B.

[cit34] Chen C. Y., Wang K., Gu L. L., Li H. (2017). RSC Adv..

[cit35] Devaraj S., Saravanakumar D., Kandaswamy M. (2007). Tetrahedron Lett..

[cit36] Zhang X., Fu J., Zhan T. G., Dai L. Y., Chen Y. Q., Zhao X. (2013). Tetrahedron Lett..

[cit37] Qian H. L., Liu C. M., Wang Z. H., Zhu D. B. (2006). Chem. Commun..

[cit38] Parthiban C., Elango K. P. (2015). Sens. Actuators, B.

[cit39] Li Y., Zheng H., Li Y., Wang S., Wu Z., Liu P., Gao Z., Liu H., Zhu D. (2007). J. Org. Chem..

[cit40] Melaimi M., Gabbaï F. P. (2005). J. Am. Chem. Soc..

[cit41] Benesi H. A., Hildebrand J. H., Benesi H., Hildebrand J. A. (1949). J. Am. Chem. Soc..

[cit42] Becke A. D. (1998). Phys. Rev. B: Condens. Matter Mater. Phys..

[cit43] Fan L., Liu Q. L., Lu D. T., Shi H. P., Yang Y. F., Li Y. F., Dong C., Shuang S. (2013). J. Mater. Chem. B.

[cit44] Ma Y. S., Li J. F., Hou S. G., Zhang J. F., Shi Z. Q., Jiang T. Y., Wei X. F. (2016). New J. Chem..

