# Supplementary material for: Novel fluorescent probes for the fluoride anion based on hydroxy-substituted perylene tetra-(alkoxycarbonyl) derivatives
Source: RSC Adv. 2018 Apr 16;8(25):14084–91. doi: 10.1039/c8ra00299a (PMC9079896; doi:10.1039/c8ra00299a)

Electronic Supplementary Material (ESI) for RSC Advances.
This journal is © The Royal Society of Chemistry 2018

Supporting Information

Novel fluorescent probes for fluoride anion based on Hydroxy-substituted perylene tetra-(alkoxycarbonyl) derivatives

Fengxia Zhang [a, b], Yunlong Zhao [a], Yanhui Chi [a], Yongshan Ma [c, d]*, Tianyi Jiang [c], Xiaofeng Wei [c], Qian Zhao [c], Zhiqiang Shi [a]* and Jingmin Shi [a]*

[a] College of Chemistry, Chemical Engineering and Materials Science, Collaborative Innovation Center of Functionalized Probes for Chemical Imaging in Universities of Shandong, Key Laboratory of Molecular and Nano Probes, Ministry of Education Shandong Provincial Key Laboratory of Clean Production of Fine Chemistry, Shandong Normal University, jinan 250014, P. R. China

[b] Shandong Provincial Key Laboratory of Metrology and Measurement, Shandong Institute of Metrology, Shandong Social Justice Institute of Metrology, Jinan 250014, P. R. China

[c] School of Municipal and Environmental Engineering, Shandong Jianzhu University, Jinan 250101，P. R. China

[d] Co-Innovation Center of Green Building, Jinan 250101，P. R. China.

* Corresponding address: mlosh@sdjzu.edu.cn, zshi@sdnu.edu.cn, shijingmin1955@163.com.

Experimental section

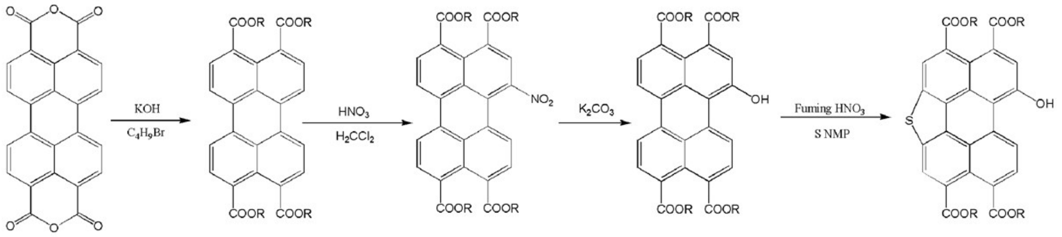

3 4 5 1 2

Scheme s-1. The synthesis route of probes **1** and **2.**

General procedure for the synthesis of 4

Compound **3** (4.0 g, 10.2 mmol) was dissolved in 200 mL KOH (0.1 mol/L) solution and heated under reflux for 6 hours. After being cooled to room temperature, the reaction mixture was added dropwise into acetone: isopropanol (1:1, v/v, 400.0 mL). The resulting precipitate was filtered and washed three times with 15mL of acetone and isopropanol, respectively. After drying, the solid was dissolved in a flask (250mL) with 100mL of water, then butyl bromide (10.88 g, 80 mmol) was added into the solution after tetrabutylammonium bromide (3.0 g, 9.4 mmol), anhydrous potassium carbonate (6.0g, 43.4g) and KI (0.125 g, 1 mmol) were charged into the solution and stirred vigorously for 15 min. The mixture was refluxed for 24 h. Subsequently, the reaction mixture was cooled to room temperature and poured into 100ml of methylene chloride, and the methylene chloride phase layer was washed thrice with 30 ml of aqueous solution. A yellow solid was obtained by adding methanol into the concentrated chloroform solution and dried under vacuum condition at 70℃. Yield: 4.58g (70%). Yellow solid. ^1H NMR (300 MHz, CDCl3) δ: 8.08 (d, J=7.9Hz, 4H), 7.93 (d, J=7.9Hz, 4H), 4.39 (d, 8H), 1.87 (m, 8H), 1.59 (m, 8H), 1.02 (m, 12H). ^{13}C NMR (75 MHz, $CDCl_3$, ppm) δ: 168.51, 132.78, 130.35, 130.26, 128.81, 126.62, 121.26, 65.29, 53.39, 30.68, 19.28, 13.78. FT-IR (KBr, cm^{-1}): ν = 2952, 2868, 2109, 1893, 1714, 1584, 1511, 1469, 1405, 1266, 1164, 1128, 1097, 1032, 939, 890, 841, 803, 743, 588, 507, 436. MALDI-TOF MS: calcd 652.3; found, 652.3046 (M + Na^+).

Elemental analysis: Calculated for $C_{40}H_{44}O_8$ C 73.60, H 6.79, O 19.61%; found C 73.48, H 6.43, O 19.82%.

General procedure for the synthesis of 5.

Compound **4** (1.0g, 1.5 mmol）was dissolved in 150mL of dichloromethane at room temperature. Fuming nitric acid (1.5mL) was then added dropwise into the solution and the reaction mixture was kept stirring for 2h at room temperature. The resulting mixture was diluted with 50 mL of sodium bicarbonate (20%) solution. The organic phase was separated, washed with water three times (50 ml×3), dried over anhydrous magnesium sulfate and concentrated under reduced pressure. The crude product was purified by gel column chromatography using dichloromethane/petroleum ether (2:1, v/v) as the eluent to afford target product. Yield: 0.9g (86%). Red solid. ^{1}H-NMR(300 MHz, $CDCl_3$, ppm): δ=8.40 (s, 1H), 8.37 (m, 2H), 8.26 (d, 1H), 8.14 (d, 1H, J=6 Hz), 7.97 (d, 1H, J=6 Hz), 7.93 (d, 1H, J=9 Hz), 4.37 (m, 8H), 1.82 (m, 8H), 1.55 (m, 8H), 1.03 (m, 12H). ^{13}C NMR (75 MHz, $CDCl_3$, ppm) δ: 168.06, 167.86, 167.79, 166.60, 146.32, 133.91, 132.53, 131.96, 131.61, 130.83, 130.51, 130.38, 130.00, 129.15, 128.73, 128.48, 127.92, 127.40, 126.63, 125.62, 123.13, 122.61, 66.02, 65.64, 65.58, 30.60, 19.23, 13.74. FT-IR (KBr, cm^{-1}): v = 2959, 2871, 1711, 1589, 1529, 1460, 1394, 1353, 1274, 1249, 1163, 1108, 1062, 1021, 959, 899, 846, 801, 736, 702, 604, 506, 434. MALDI-TOF MS: calcd 697.29; found, 697.2913 (M + Na^+). Elemental analysis: Calculated for $C_{40}H_{43}NO_{10}$ C 68.85, H 6.21, N 2.01, O 22.93%; found C 68.75, H 6.33, O 2.04, O 22.87%.

General procedure for the synthesis of 1.

Compound **5** (70mg, 0.1mmol), potassium carbonate (69 mg, 0.50 mmol) were dissolved in N-methylpyrrolidone (NMP, 10ml). The resulting solution was heated to 60℃ with vigorous stirring for 12h, then cooled and poured into 100ml of 2 M HCl. The precipitate was collected by vacuum filtration, washed with water and dried under vacuum condition. The residue was purified by column chromatography on silica gel with dichloromethane /ethyl acetate (20/1) as eluent and a carmine solid of was obtained. Yield: 52 mg (79%). Red solid. ^{1}H-NMR ($CHCl_3$, TMS, ppm): δ =

10.67 (s, 1H), 9.34 (d, 1H), 8.25 (d, 1H), 7.99-7.95 (m, 3H), 7.84 (d, 1H), 7.82 (s, 1H), 4.23-4.17(m, 8H), 2.62-2.39(m, 8H), 1.71-1.64(m, 8H), 1.44-1.34(m, 12H). ^{13}C NMR (75 MHz, $CDCl_3$, ppm): δ = 168.78, 167.80, 167.71, 157.31, 144.10, 133.47, 132.33, 131.95, 131.82, 129.85, 129.30, 128.61, 127.65, 127.40, 126.34, 125.87, 125.64, 122.24, 120.78, 113.56, 80.00, 65.35, 65.27, 65.21, 30.66, 30.58, 19.25, 13.77. FT-IR (KBr, cm^{-1}): v = 2957, 2928, 2870, 1708, 1588, 1514, 1460, 1406, 1344, 1271, 1196, 1160, 1063, 1024, 961, 939, 896, 837, 801, 750, 707, 580, 507, 438. MALDI-TOF MS: calcd 667.3; found, 667.29 (M + Na^+). Elemental analysis: Calculated for $C_{40}H_{44}O_9$ C 71.84, H 6.63, O 21.53%; found C 71.76, H 6.25, O 21.61%.

General procedure for the synthesis of 2.

Compound **1** (335 mg, 0.5 mmol) was dissolved in 50mL of dichloromethane at room temperature. Fuming nitric acid (2.5 mL) was then added dropwise into the solution and the reaction mixture was kept stirring for 1h at room temperature. The resulting mixture was diluted with 100 mL of sodium bicarbonate (20%) solution. The organic phase was separated, washed with water three times (50 ml×3), dried over anhydrous $MgSO_4$ and concentrated under reduced pressure. Then a mixture of the crude product (250mg, 0.4mmol) and sulfur powder (160mg 5.0mmol) was dissolved in 150mL anhydrous N-methylpyrrolidine. The resulting solution was heated to 110℃ with vigorous stirring for 5h, then cooled and poured into 200ml of 2 M HCl. The precipitate was collected by vacuum filtration, washed with water three times (50 ml×3), and dried under vacuum condition. The residue was purified by gel column chromatography with dichloromethane /ethyl acetate (20/1) as eluent to afford target product **2 (**196mg, 82%). Characterization data: ^{1}H-NMR ($CDCl_3$, 300 MHz, ppm): δ = 11.09 (s, 1H), 9.73 (d, J = 9.0 Hz, 1H), 8.71 (s, 1H), 8.55 (s, 1H), 8.33 (s, 1H), 8.11 (d, 1H), 4.34-4.43 (m, 8), 1.74 (m, 8H), 1.48 (m, 8H), 0.88-0.99 (m, 12H). ^{13}C NMR (75 MHz, CDCl3, ppm): δ = 169.59, 169.20, 168.81, 153.19, 129.87, 129.08, 128.16, 127.77, 126.94, 126.77, 125.43, 124.15, 123.01, 122.27, 119.10, 116.71, 65.71, 65.61, 65.42, 30.81, 29.67, 19.34, 13.84. FT-IR (KBr, cm^{-1}): v = 3365 (s, O-H stretching),

3189 (s, aliphatic C-H), 2922 (vs, aliphatic C-H), 2854 (vs, aliphatic C-H), 1651 (s, C=O), 1462 (s, aromatic C=C), 1410 (s, C-O), 1067 (s, C-C), 873 (s, C-S), 732, 661, 586, 528, 486, 437. HRMS: $C_{40}H_{42}SO_9$ (M^+ -H), calcd, 697.2352, found 697.2469.

Spectra of NMR, FTIR and MALDI-TOF-MS

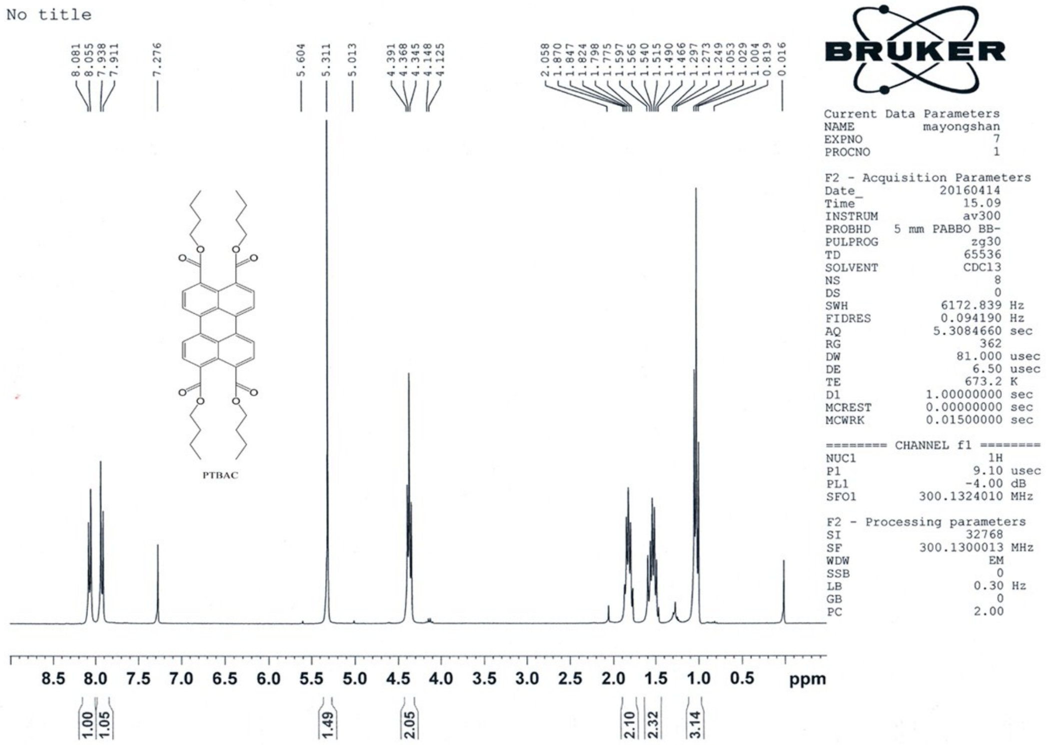

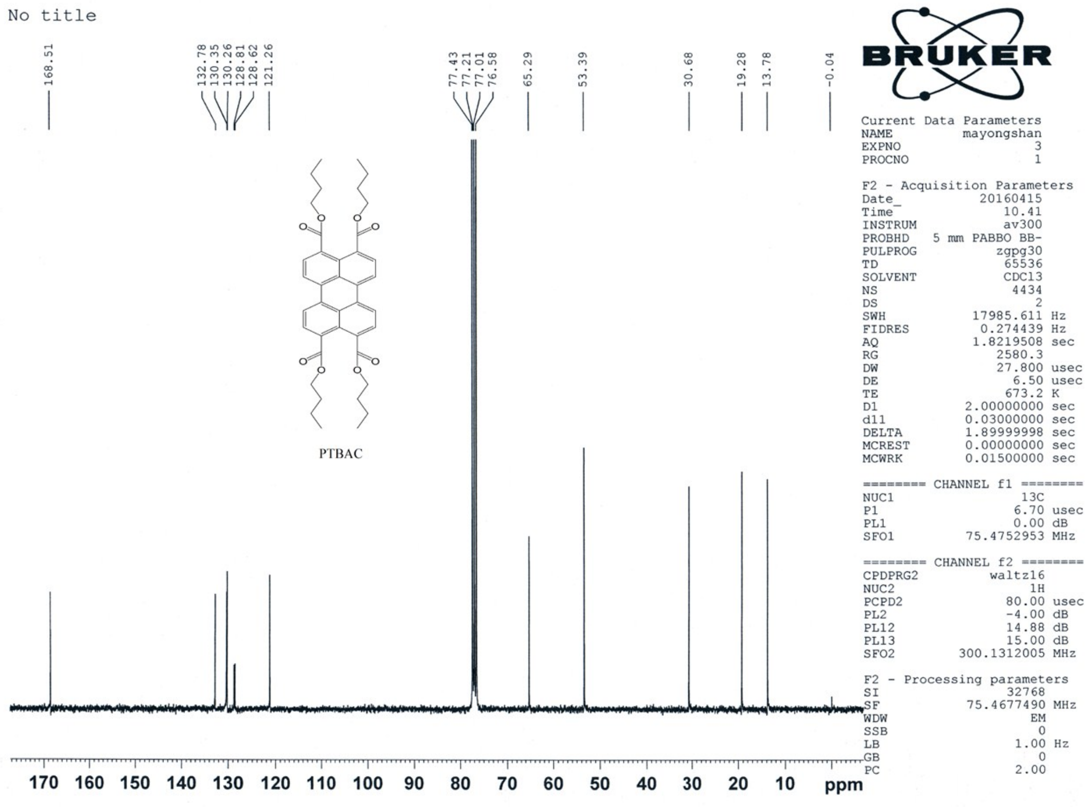

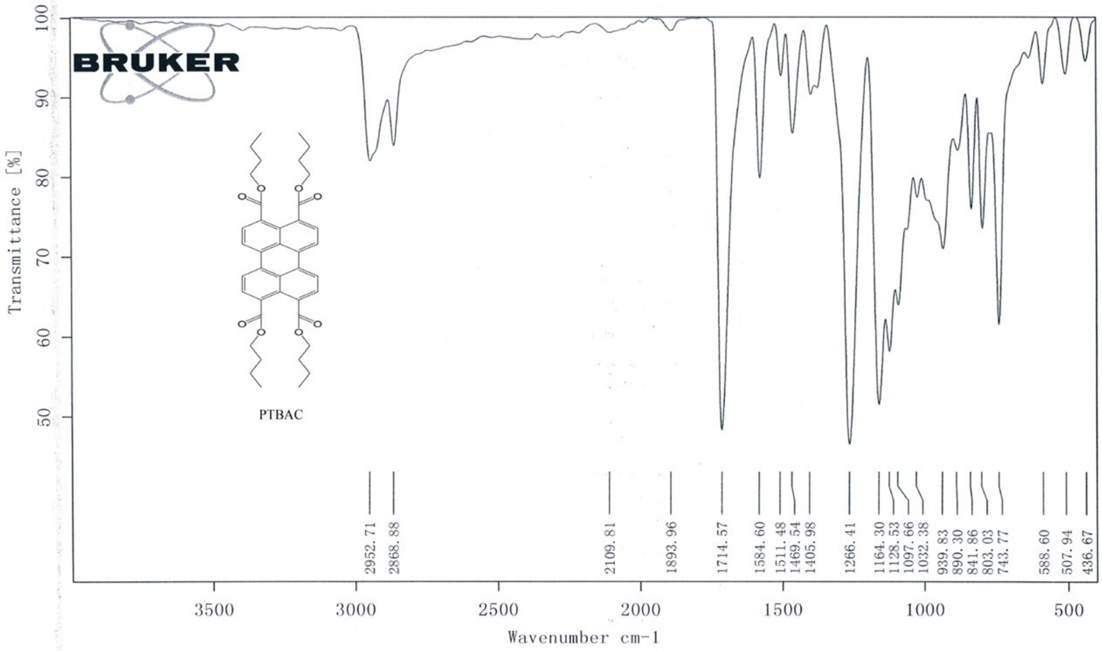
BRUKER
Transmittance [%]
100
90
80
70
60
50
PTBAC
2952.71
2868.88
2109.81
1893.96
1714.57
1584.60
1511.48
1469.54
1405.98
1266.41
1164.30
1128.53
1097.66
1032.38
939.83
890.30
841.86
803.03
743.77
588.60
507.94
436.67
3500
3000
2500
2000
1500
1000
500
Wavenumber cm-1

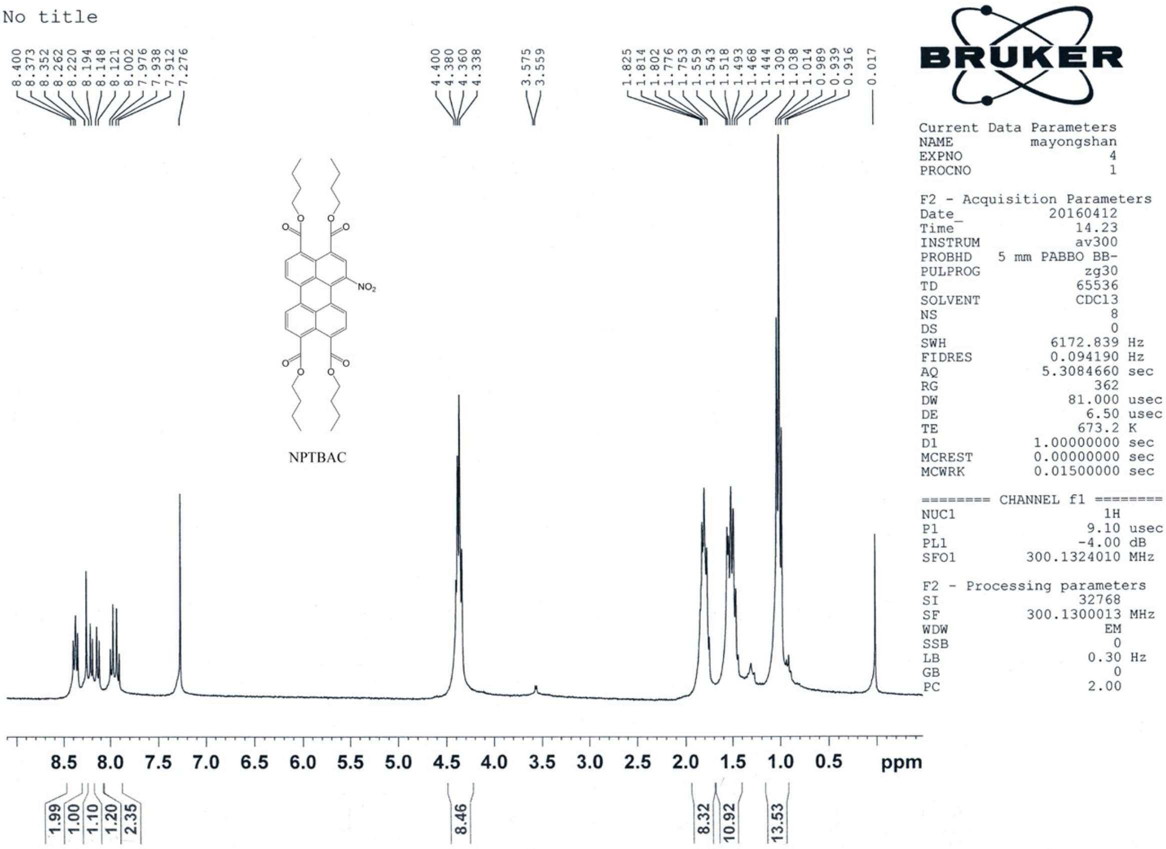
No title
8.400
8.373
8.352
8.262
8.220
8.194
8.148
8.121
8.002
7.976
7.938
7.912
7.276
4.400
4.380
4.360
4.338
3.575
3.559
1.825
1.814
1.802
1.776
1.753
1.559
1.543
1.518
1.493
1.468
1.444
1.309
1.038
1.014
0.989
0.939
0.916
0.017
NO2
NPTBAC
BRUKER
Current Data Parameters
NAME mayongshan
EXPNO 4
PROCNO 1
F2 - Acquisition Parameters
Date_ 20160412
Time 14.23
INSTRUM av300
PROBHD 5 mm PABBO BB-
PULPROG zg30
TD 65536
SOLVENT CDCl3
NS 8
DS 0
SWH 6172.839 Hz
FIDRES 0.094190 Hz
AQ 5.3084660 sec
RG 362
DW 81.000 usec
DE 6.50 usec
TE 673.2 K
D1 1.00000000 sec
MCREST 0.00000000 sec
MCWRK 0.01500000 sec
======== CHANNEL f1 ========
NUC1 1H
P1 9.10 usec
PL1 -4.00 dB
SFO1 300.1324010 MHz
F2 - Processing parameters
SI 32768
SF 300.1300013 MHz
WDW EM
SSB 0
LB 0.30 Hz
GB 0
PC 2.00
8.5 8.0 7.5 7.0 6.5 6.0 5.5 5.0 4.5 4.0 3.5 3.0 2.5 2.0 1.5 1.0 0.5 ppm
1.99
1.00
1.10
1.20
2.35
8.46
8.32
10.92
13.53

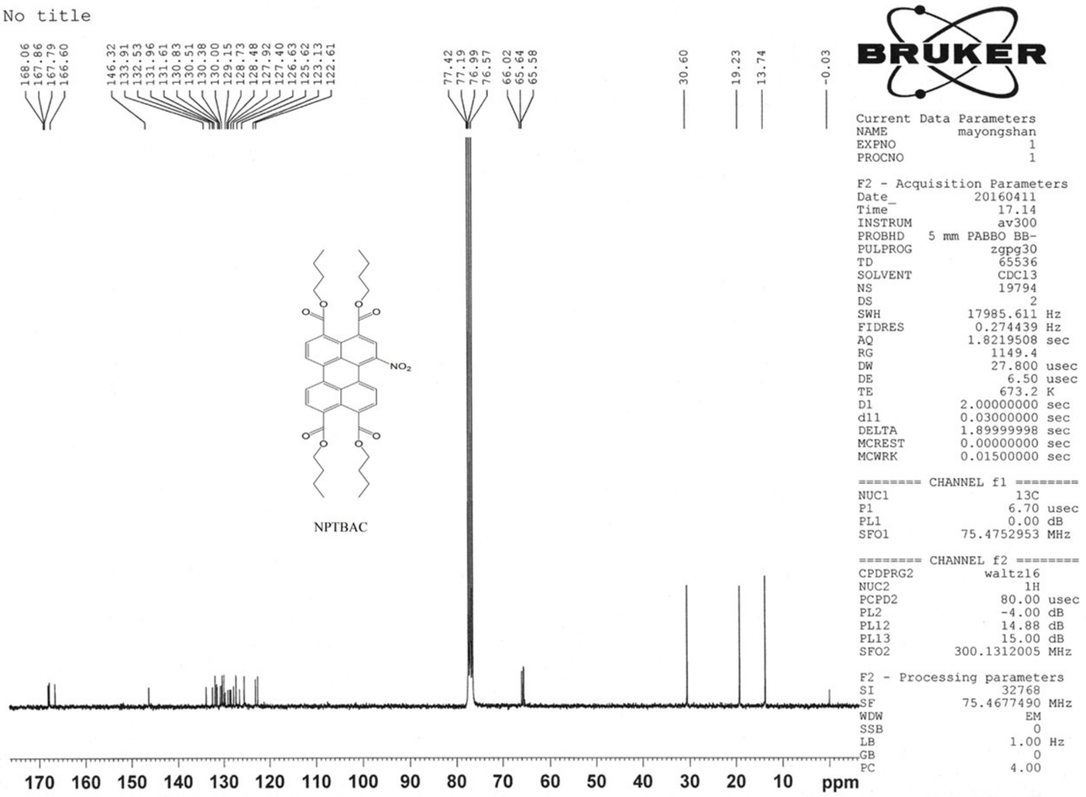
No title
168.06
167.86
167.79
166.60
146.32
133.91
132.53
131.96
131.61
130.83
130.51
130.38
130.00
129.15
128.73
128.48
127.92
127.40
126.63
125.62
123.13
122.61
77.42
77.19
76.99
76.57
66.02
65.64
65.58
30.60
19.23
13.74
-0.03
BRUKER
Current Data Parameters
NAME mayongshan
EXPNO 1
PROCNO 1
F2 - Acquisition Parameters
Date_ 20160411
Time 17.14
INSTRUM av300
PROBHD 5 mm PABBO BB-
PULPROG zgpg30
TD 65536
SOLVENT CDCl3
NS 19794
DS 2
SWH 17985.611 Hz
FIDRES 0.274439 Hz
AQ 1.8219508 sec
RG 1149.4
DW 27.800 usec
DE 6.50 usec
TE 673.2 K
D1 2.00000000 sec
d11 0.03000000 sec
DELTA 1.89999998 sec
MCREST 0.00000000 sec
MCWRK 0.01500000 sec
======== CHANNEL f1 ========
NUC1 13C
P1 6.70 usec
PL1 0.00 dB
SFO1 75.4752953 MHz
======== CHANNEL f2 ========
CPDPRG2 waltz16
NUC2 1H
PCPD2 80.00 usec
PL2 -4.00 dB
PL12 14.88 dB
PL13 15.00 dB
SFO2 300.1312005 MHz
F2 - Processing parameters
SI 32768
SF 75.4677490 MHz
WDW EM
SSB 0
LB 1.00 Hz
GB 0
PC 4.00
NO2
NPTBAC
170 160 150 140 130 120 110 100 90 80 70 60 50 40 30 20 10 ppm

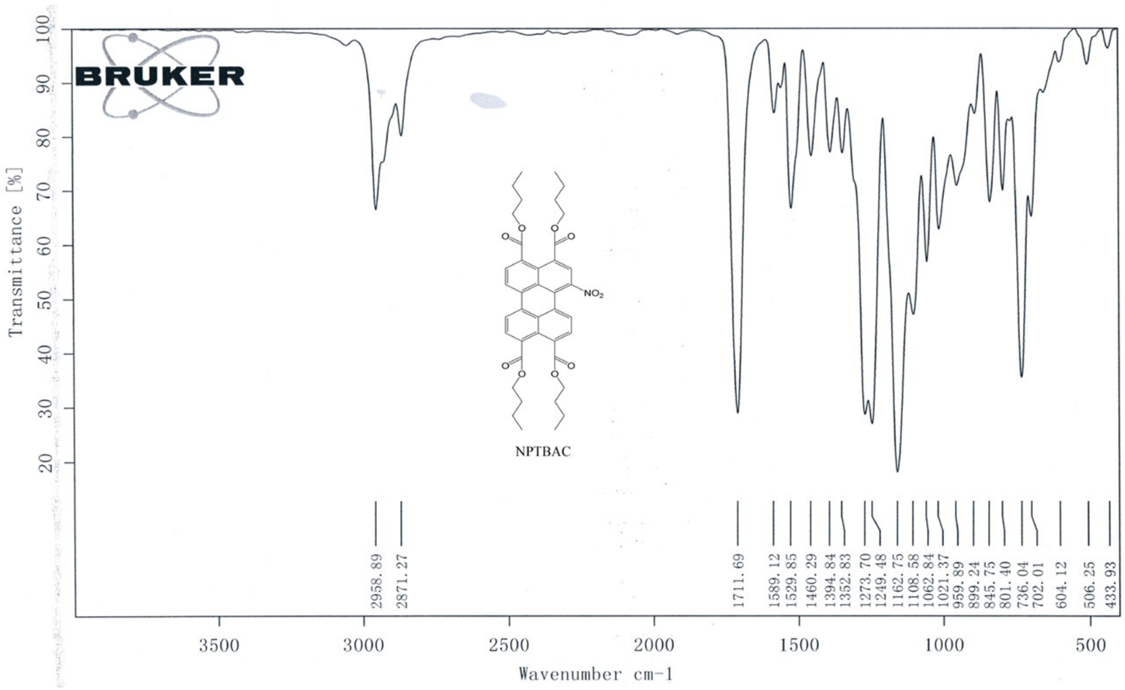
BRUKER
Transmittance [%]
100 90 80 70 60 50 40 30 20
NO2
NPTBAC
2958.89
2871.27
1711.69
1589.12
1529.85
1460.29
1394.84
1352.83
1273.70
1249.48
1162.75
1108.58
1062.84
1021.37
959.89
899.24
845.75
801.40
736.04
702.01
604.12
506.25
433.93
3500 3000 2500 2000 1500 1000 500
Wavenumber cm-1

No title

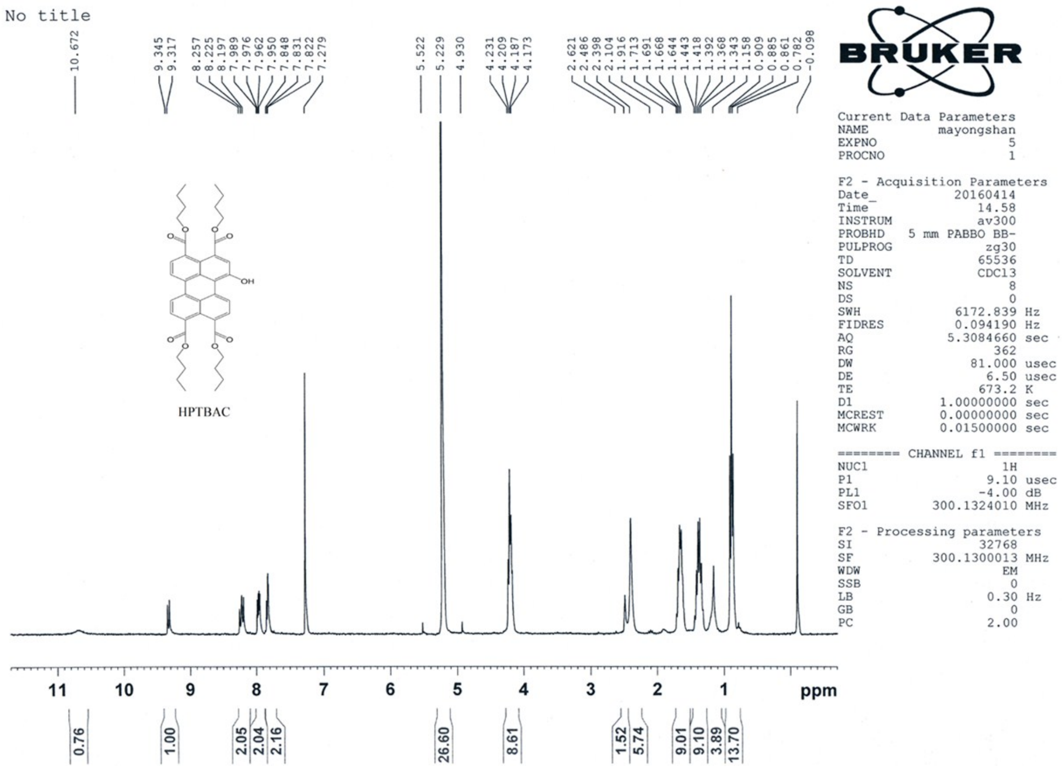
10.672
9.345
9.317
8.257
8.225
8.197
7.989
7.976
7.962
7.950
7.848
7.831
7.822
7.279
5.522
5.229
4.930
4.231
4.209
4.187
4.173
2.621
2.486
2.398
2.104
1.916
1.713
1.691
1.668
1.644
1.443
1.418
1.392
1.368
1.343
1.158
0.909
0.885
0.861
0.782
-0.098
OH
HPTBAC
BRUKER
Current Data Parameters
NAME mayongshan
EXPNO 5
PROCNO 1
F2 - Acquisition Parameters
Date_ 20160414
Time 14.58
INSTRUM av300
PROBHD 5 mm PABBO BB-
PULPROG zg30
TD 65536
SOLVENT CDCl3
NS 8
DS 0
SWH 6172.839 Hz
FIDRES 0.094190 Hz
AQ 5.3084660 sec
RG 362
DW 81.000 usec
DE 6.50 usec
TE 673.2 K
D1 1.00000000 sec
MCREST 0.00000000 sec
MCWRK 0.01500000 sec
======== CHANNEL f1 ========
NUC1 1H
P1 9.10 usec
PL1 -4.00 dB
SFO1 300.1324010 MHz
F2 - Processing parameters
SI 32768
SF 300.1300013 MHz
WDW EM
SSB 0
LB 0.30 Hz
GB 0
PC 2.00
11 10 9 8 7 6 5 4 3 2 1 ppm
0.76
1.00
2.05
2.04
2.16
26.60
8.61
1.52
5.74
9.01
9.10
3.89
13.70

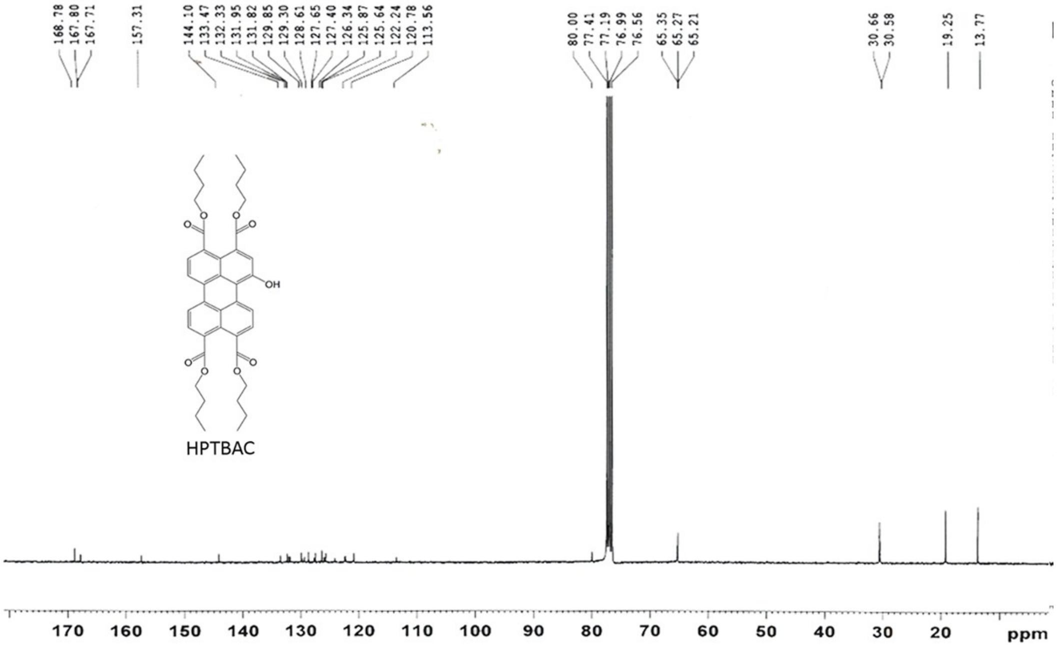
168.78
167.80
167.71
157.31
144.10
133.47
132.33
131.95
131.82
129.85
129.30
128.61
127.65
127.40
126.34
125.87
125.64
122.24
120.78
113.56
80.00
77.41
77.19
76.99
76.56
65.35
65.27
65.21
30.66
30.58
19.25
13.77
OH
HPTBAC
170 160 150 140 130 120 110 100 90 80 70 60 50 40 30 20 ppm

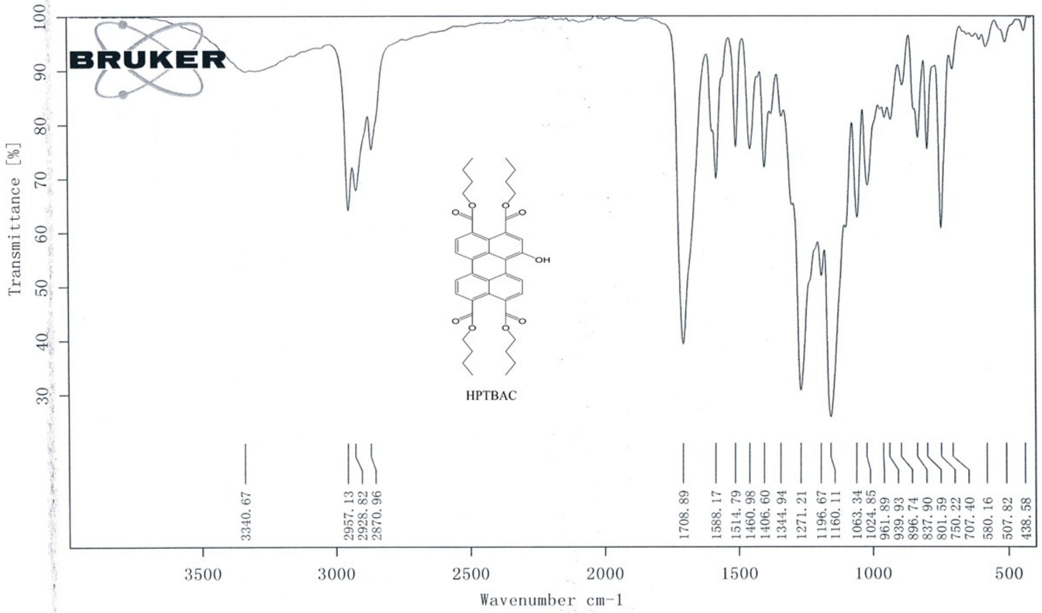
BRUKER
Transmittance [%]
Wavenumber cm-1
HPTBAC
OH
3340.67
2957.13
2928.82
2870.96
1708.89
1588.17
1514.79
1460.98
1406.60
1344.94
1271.21
1196.67
1160.11
1063.34
1024.85
961.89
939.93
896.74
837.90
801.59
750.22
707.40
580.16
507.82
438.58
3500
3000
2500
2000
1500
1000
500
100
90
80
70
60
50
40
30

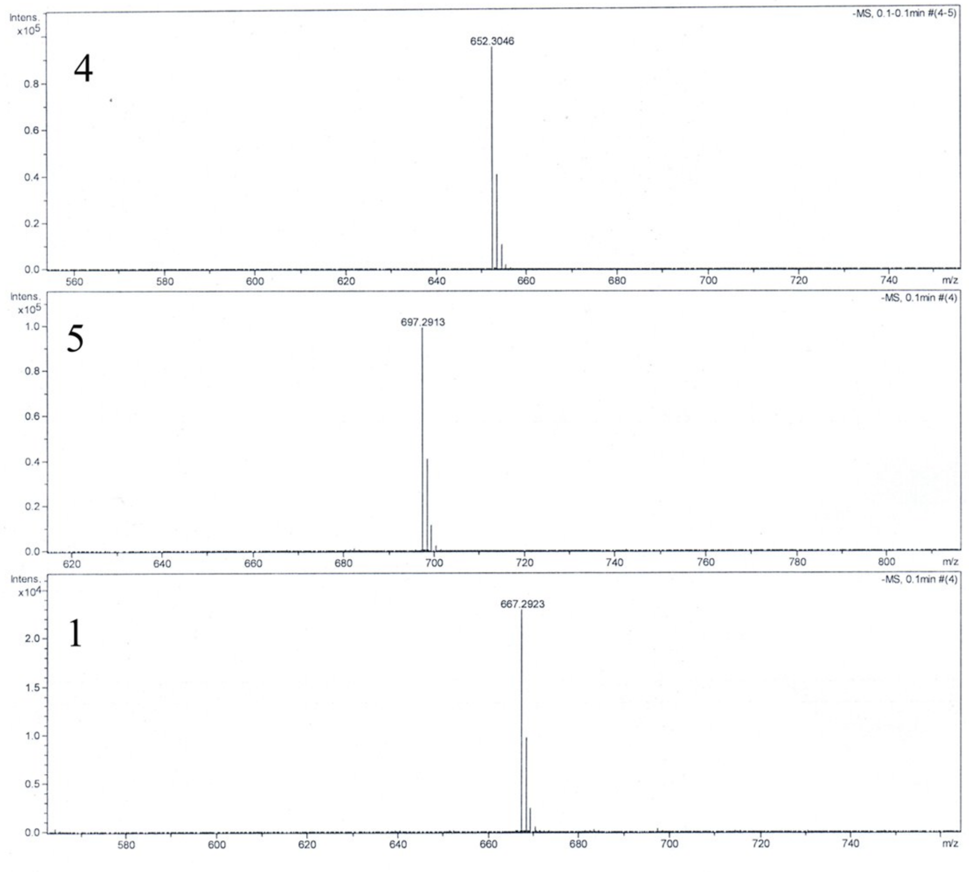
Intens.
x10^5
-MS, 0.1-0.1min #(4-5)
4
652.3046
m/z
Intens.
x10^5
-MS, 0.1min #(4)
5
697.2913
m/z
Intens.
x10^4
-MS, 0.1min #(4)
1
667.2923
m/z

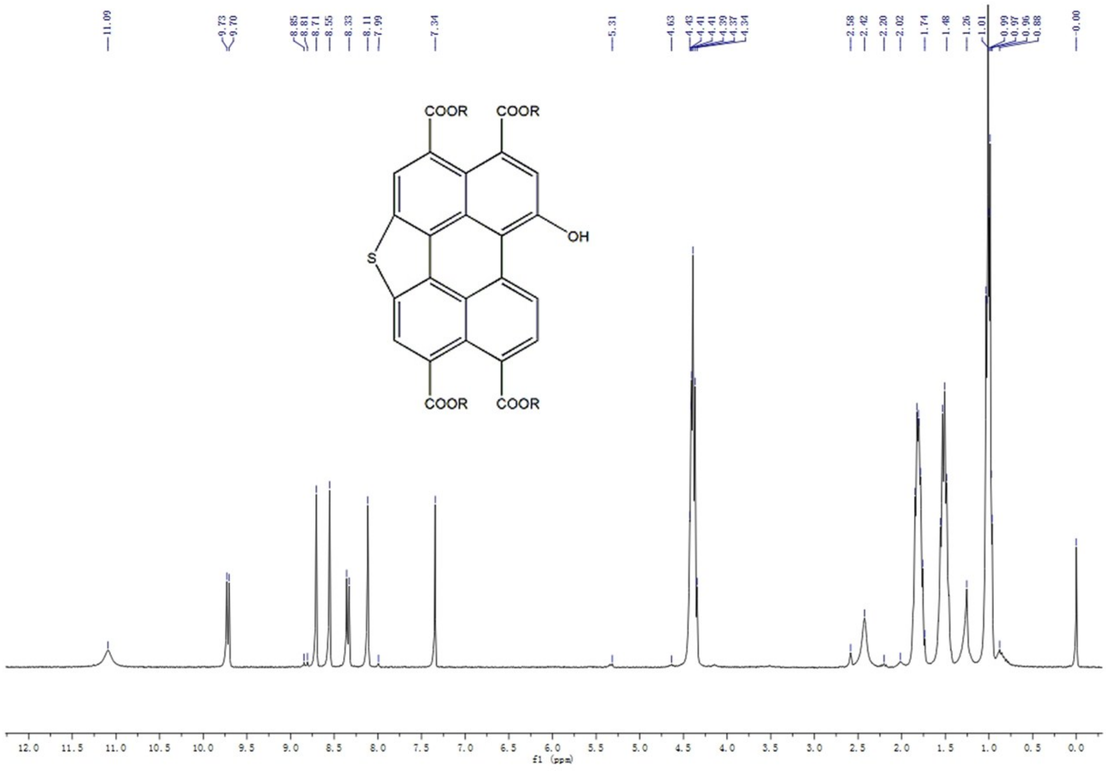
11.09
9.73
9.70
8.85
8.81
8.71
8.55
8.33
8.11
7.99
7.34
5.31
4.63
4.43
4.41
4.41
4.39
4.37
4.34
2.58
2.42
2.20
2.02
1.74
1.48
1.26
1.01
0.99
0.97
0.96
0.88
0.00
COOR
COOR
OH
S
COOR
COOR
f1 (ppm)

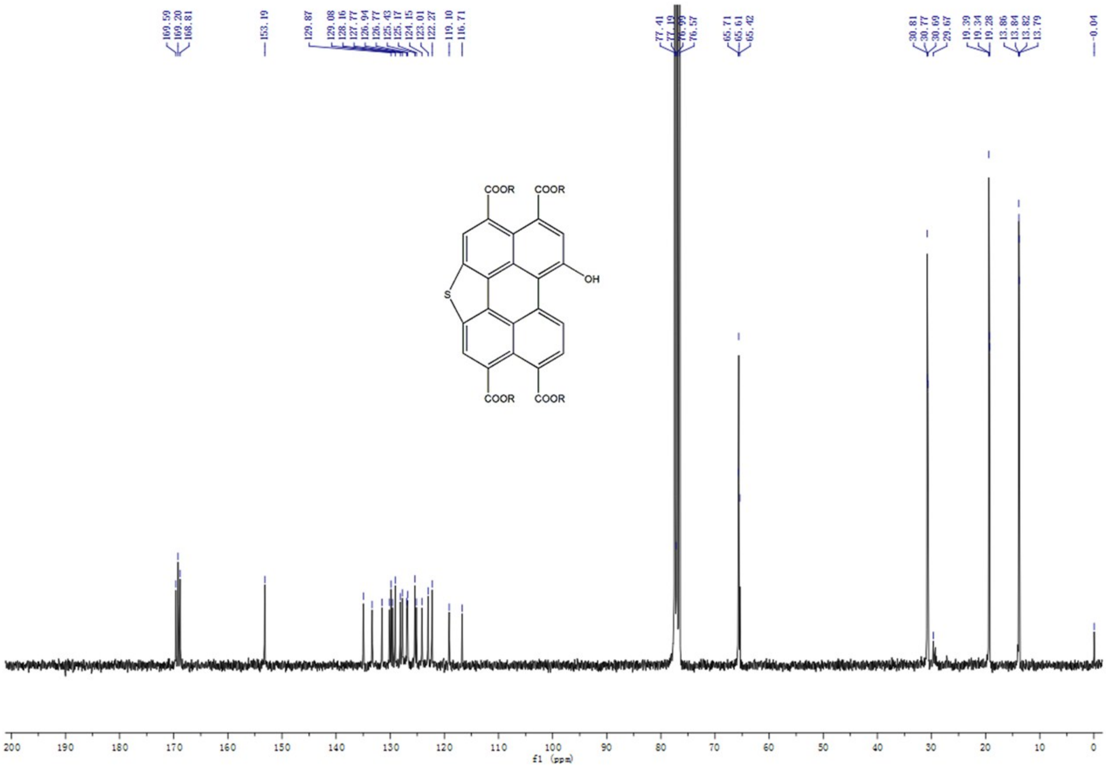
169.59
169.20
168.81
153.19
129.87
129.08
128.16
127.77
126.94
126.77
125.43
125.17
124.15
123.01
122.27
119.10
116.71
77.41
77.19
76.99
76.57
65.71
65.61
65.42
30.81
30.77
30.69
29.67
19.39
19.34
19.28
13.86
13.84
13.82
13.79
0.04
COOR
COOR
OH
S
COOR
COOR
f1 (ppm)

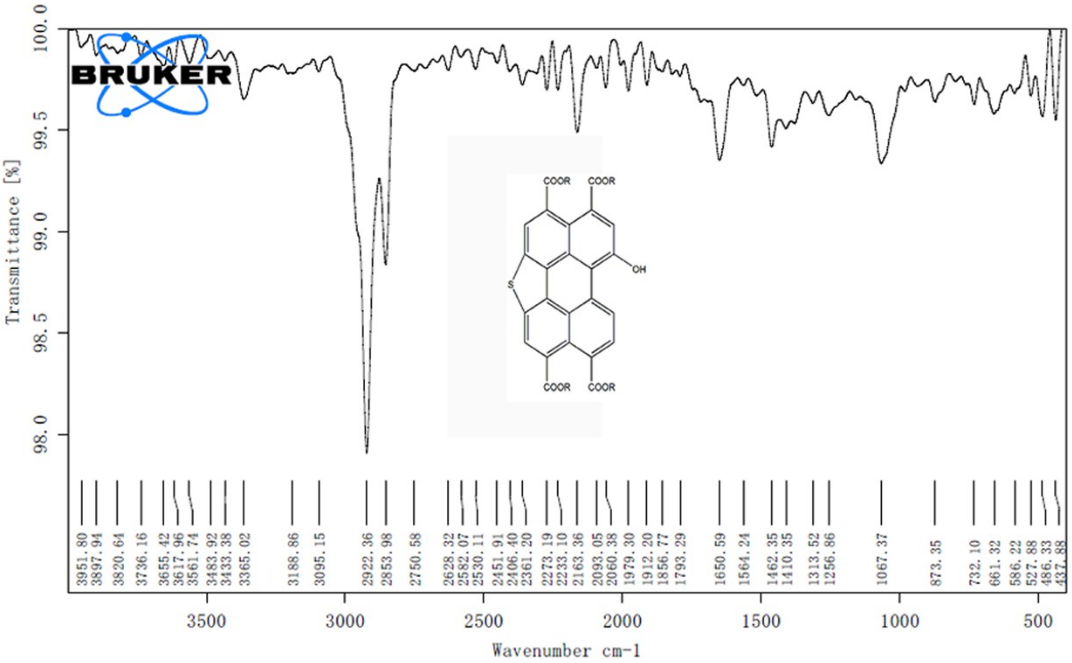

Transmittance [%]
100.0
99.5
99.0
98.5
98.0
BRUKER
COOR
COOR
OH
S
COOR
COOR
3951.80
3897.94
3820.64
3736.16
3655.42
3617.96
3561.74
3483.92
3433.38
3365.02
3188.86
3095.15
2922.36
2853.98
2750.58
2628.32
2582.07
2530.11
2451.91
2406.40
2361.20
2273.19
2233.10
2163.36
2093.05
2060.38
1979.30
1912.20
1856.77
1793.29
1650.59
1564.24
1462.35
1410.35
1313.52
1256.86
1067.37
873.35
732.10
661.32
586.22
527.88
486.33
437.88
3500
3000
2500
2000
1500
1000
500
Wavenumber cm-1

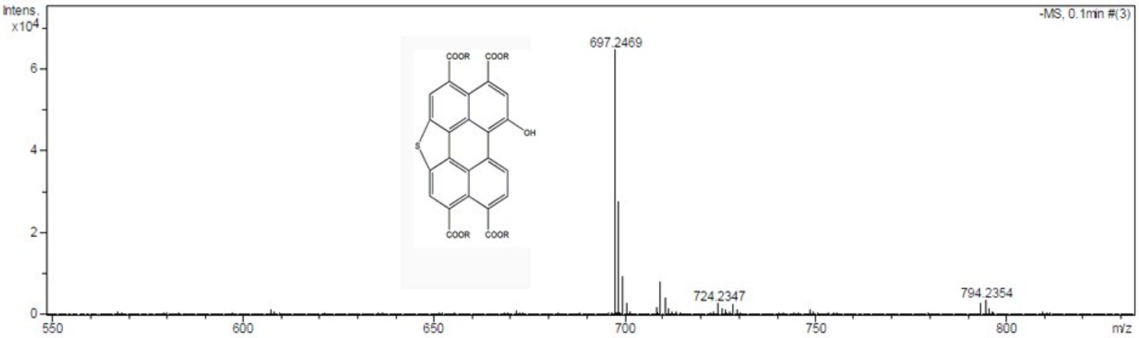

Intens.
x10^4
-MS, 0.1min #(3)
6
4
2
0
697.2469
724.2347
794.2354
COOR
COOR
OH
S
COOR
COOR
550
600
650
700
750
800
m/z

Fig. S-1 Job's plot for probes **1** and **2** with F^- ions.

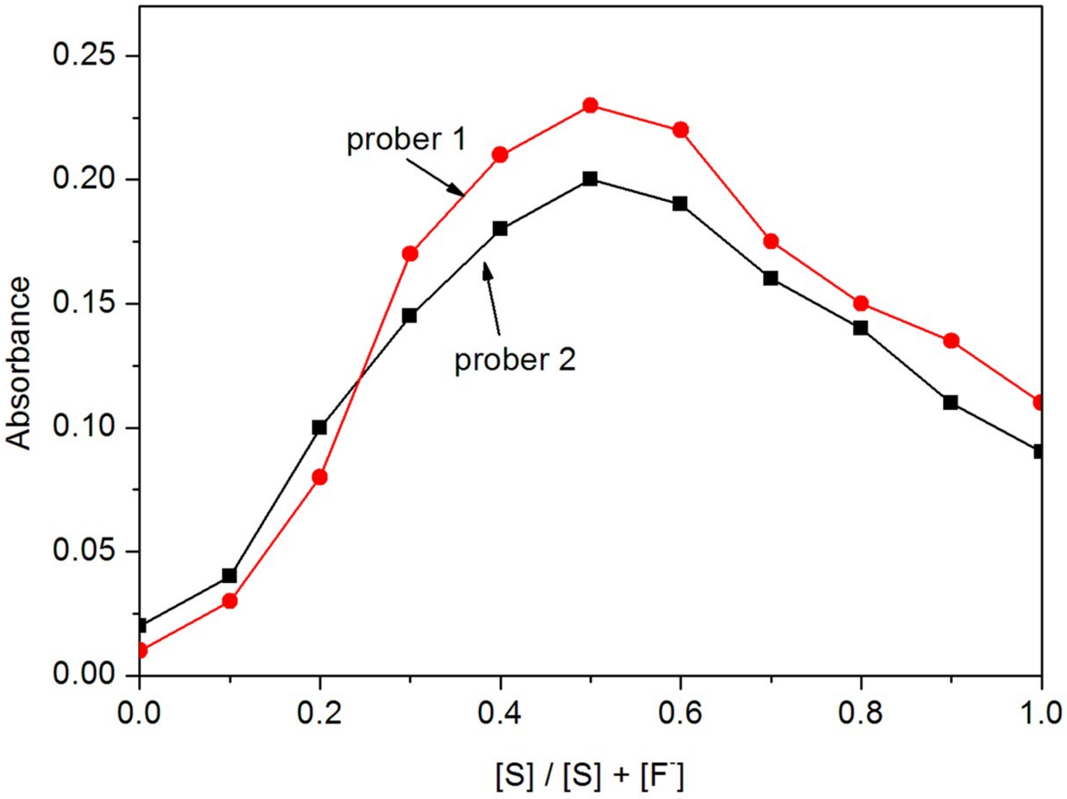

Fig. S-2 Plot of (a) the emission intensity of sensor **1** at 517 nm, (b) the emission intensity of sensor **2** at 475 nm vs concentration of F^- in DCM.

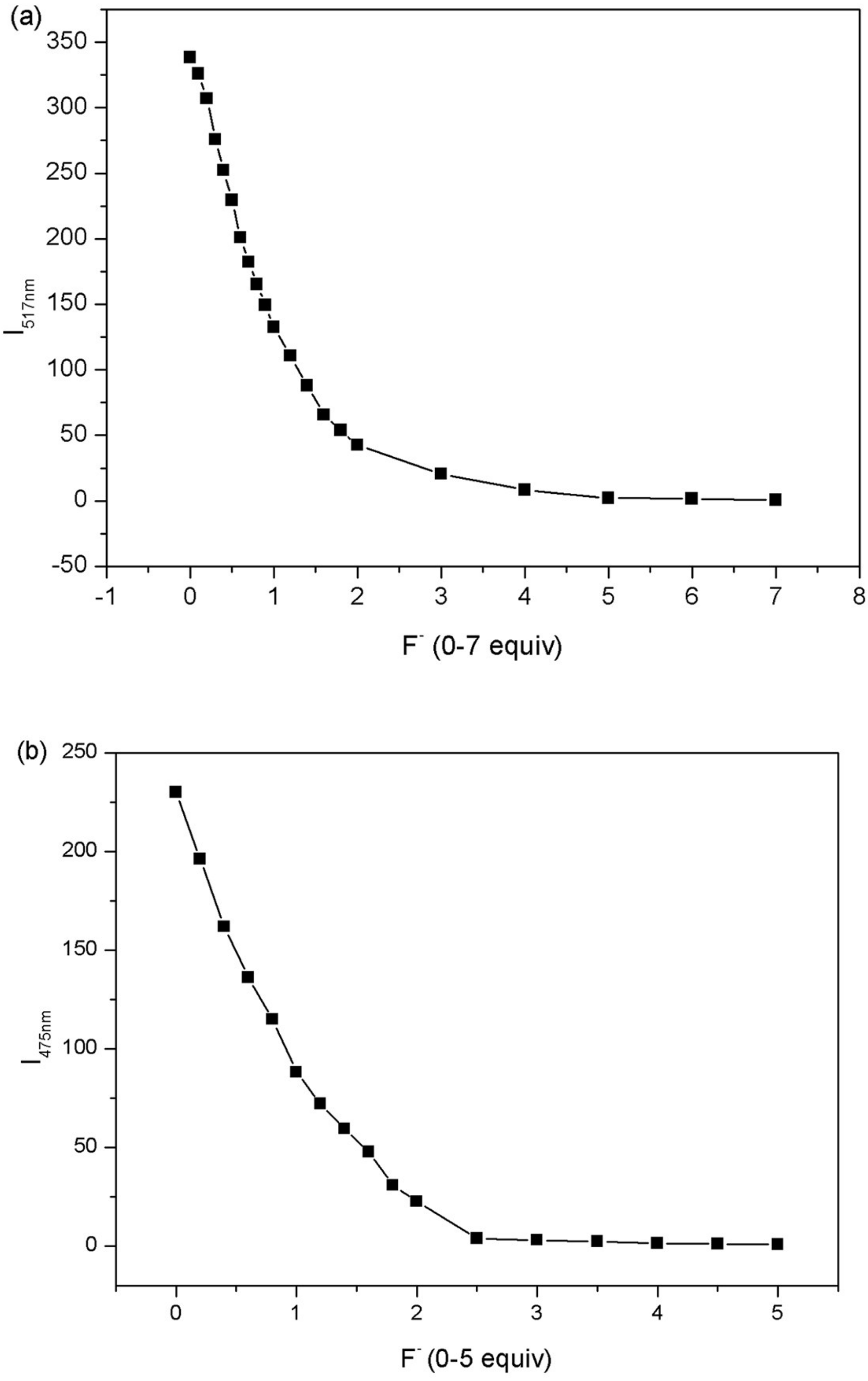

Fig. S-3 Cell cytotoxic effect of (a) probes **1** and (b) **2** on Human Lung Cancer A549 Cells. (1, control; 2, 0.01 μM; 3, 0.1μM; 4, 1 μM; 5, 10 μM; 6, 50 μM. Data are expressed as mean values standard error of the mean of five independent experiments).

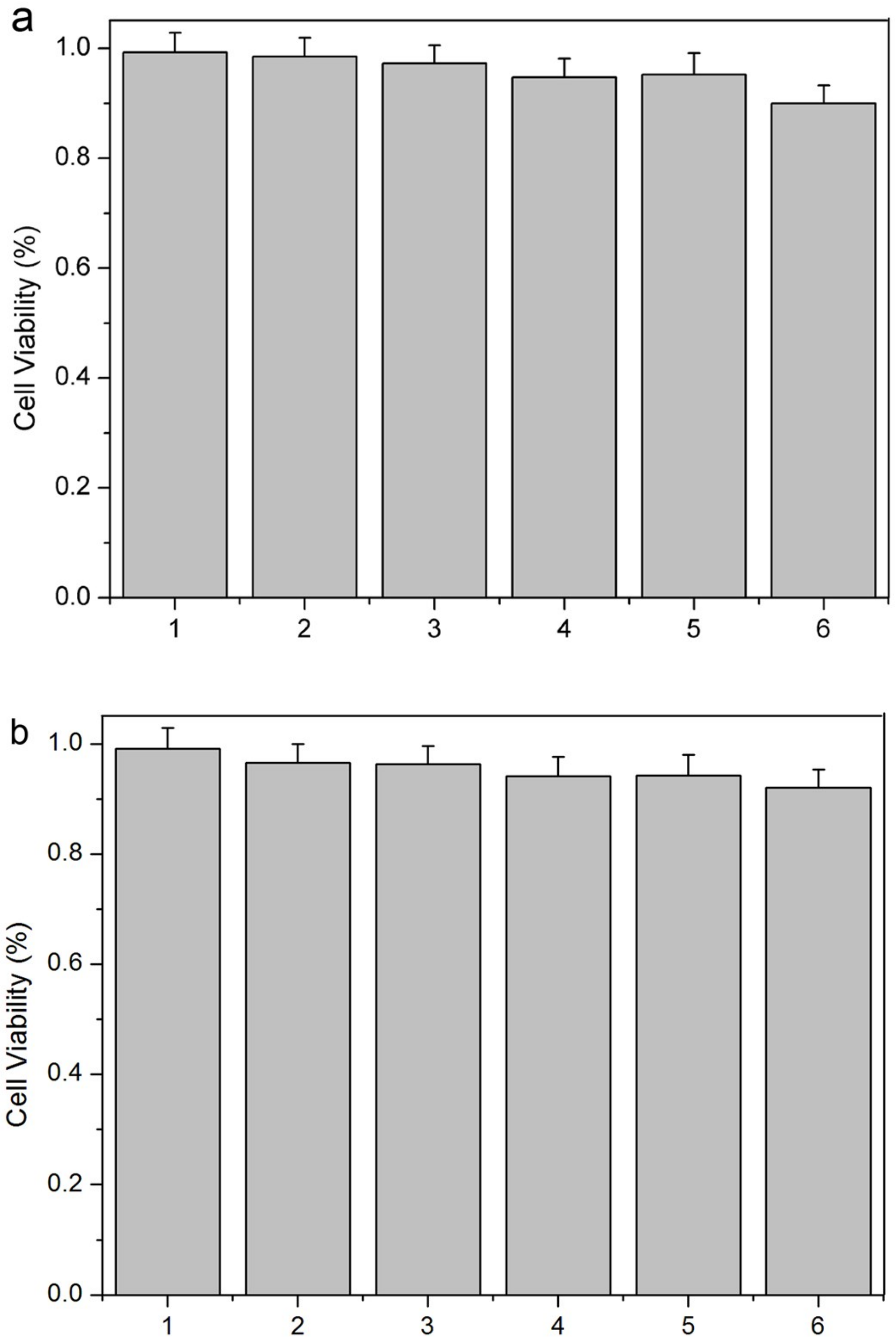

Supplement: RA-008-C8RA00299A-s001 [file RA-008-C8RA00299A-s001.pdf]
